# On the Interactions of Melatonin/β-Cyclodextrin Inclusion Complex: A Novel Approach Combining Efficient Semiempirical Extended Tight-Binding (xTB) Results with Ab Initio Methods

**DOI:** 10.3390/molecules26195881

**Published:** 2021-09-28

**Authors:** Riccardo Ferrero, Stefano Pantaleone, Massimo Delle Piane, Fabrizio Caldera, Marta Corno, Francesco Trotta, Valentina Brunella

**Affiliations:** 1 Dipartimento di Chimica and Nanostructured Interfaces and Surfaces (NIS) Centre, Università degli Studi di Torino, via P. Giuria 7, IT-10125 Torino, Italy; riccardo.ferrero@unito.it (R.F.); stefano.pantaleone@unito.it (S.P.); fabrizio.caldera@unito.it (F.C.); francesco.trotta@unito.it (F.T.); 2 Dipartimento di Chimica, Biologia e Biotecnologie, Università degli Studi di Perugia, via Elce di Sotto 8, I-06123 Perugia, Italy; 3 Politecnico di Torino, Department of Applied Science and Technology (DISAT), Corso Duca degli Abruzzi, 24, 10129 Torino, Italy; massimo.dellepiane@polito.it

**Keywords:** melatonin, β-cyclodextrin, inclusion complex, DFT, molecular dynamics, drug-delivery system

## Abstract

Melatonin (MT) is a molecule of paramount importance in all living organisms, due to its presence in many biological activities, such as circadian (sleep–wake cycle) and seasonal rhythms (reproduction, fattening, molting, etc.). Unfortunately, it suffers from poor solubility and, to be used as a drug, an appropriate transport vehicle has to be developed, in order to optimize its release in the human tissues. As a possible drug-delivery system, β-cyclodextrin (βCD) represents a promising scaffold which can encapsulate the melatonin, releasing when needed. In this work, we present a computational study supported by experimental IR spectra on inclusion MT/βCD complexes. The aim is to provide a robust, accurate and, at the same time, low-cost methodology to investigate these inclusion complexes both with static and dynamic simulations, in order to study the main actors that drive the interactions of melatonin with β-cyclodextrin and, therefore, to understand its release mechanism.

## 1. Introduction

Melatonin (N-acetyl-5-methoxytryptamine, MT) is a ubiquitous molecule, present in all living organisms [1], produced in the pineal gland and secreted as a hormone. Indeed, in humans, MT is involved in the regulation of various neuroendocrine physiologic cellular processes [2] and has a wide range of beneficial effects. Firstly, it is directly involved in the circadian cycle, in order to induce the correct sleep–wake cycle [3,4,5]. Melatonin has also been recently studied as a powerful inhibiter of free radicals, able to prevent, at least partially, aging [6,7]. Finally, recent publications assign to melatonin an important role as a therapeutic application for Alzheimer disease [8], cardiac disease, cancer and as an immunomodulator [5].

However, its employment is limited by a low water solubility (0.002 mol/kg) [9] and by its relative sensitivity to light [10]. For these reasons, the studies in the last years have turned towards the creation of a drug-delivery system (DDS) between melatonin and β-cyclodextrin [11,12,13], based on their host–guest interaction, maximized with the use of cyclodextrins with seven units of α-D-glucose.

Cyclodextrins feature a hydrophilic outer surface and non-polar cavity that enable melatonin to form complexes through the insertion of the hydrophobic portion of the molecule inside the non-polar cavity. Cyclodextrins (CDs) are cyclic oligosaccharides composed of different α-D-glucose units [14]. The three more important CDs are the α-, β- and γ-cyclodextrin, respectively formed by six, seven and eight single units of glucose. All of these show a toroidal symmetry, making it like a truncated hollow cone, with different molecular dimensions depending on the specific type of cyclodextrin [15,16].

CDs are chemical compounds with a marked tendency to form inclusion complexes with a variety of substrates. In fact, host–guest phenomena regarding inclusion complexes formed through non-covalent interactions are important in different disciplines, such as chemistry, biology and pharmacy [17]. Most of the pharmacologically active molecules have comparable molecular sizes, compatible with the dimension of the β-cyclodextrin cavity. With a height of 7.8 Å and a diameter of the internal cavity of about 7.5 Å [18], β-cyclodextrin is often the perfect host partner for a certain number of guest molecules of biological interest [19,20,21,22,23,24,25,26,27]. 

Previous studies investigated the formation of non-covalent complexes between a guest and β-cyclodextrin with different computational methods and at different levels of theory. These kinds of complexes have been computationally investigated since the end of the XX century, treated at the classical mechanics or semi-empirical level [18,28,29,30,31], usually used as a complementary tool to experimental techniques, such as NMR [26,32,33,34,35,36], fluorescence [37] and X-ray diffraction [35,36], depending on the nature of the guest molecule. More recently, DFT methods have become the method of choice in computational chemistry because, at a reasonable computational cost, they are able to provide accurate geometrical parameters, properties, such as IR spectra, and complexation energies for a wide variety of systems, including organic systems. Therefore, they were used extensively to study these host–guest partners [20,22,38,39,40], often accompanied by experimental techniques, showing a good agreement, in particular between experimental and calculated IR spectra [21,24,41]. In recent years, to increase the complexity and realism of the studied systems, DFT methods have been often replaced by cheaper semi-empirical and force-field-based methods. Modern force-fields are used to perform large-scale molecular dynamics simulations [42,43], whose cost at the DFT level would be too high. 

A new candidate, aimed at bridging the gap between the accuracy of DFT and the speed of semi-empirical methods, which received particular attention for a more efficient and less expensive treatment of a general system, is the recently developed semiempirical xTB-GFN2 method by Grimme’s group. Based on a revised version of Tight-Binding methodology (and on the novel D4 London dispersion correction), it is capable to provide accurate results at a reduced computational cost, orders of magnitude lower with respect to DFT. It has been recently employed in a large variety of systems, also including substrates different from cyclodextrin, demonstrating a good accuracy, comparable with DFT [44,45,46]. In addition, also xTB-GFNFF, based on a completely automated partially polarizable generic force-field, has been developed. This particular force-field has the great advantage that it does not require any information about the topology of the system, but only the coordinate file in XYZ format, and, accordingly, this method does not need any pre-production procedure.

In the present paper, we use inclusion MT/βCD complexes as a testing ground for these novel methods, validating them against more standard DFT-based approaches, in terms of structure, energetics and IR spectra prediction. The aim is to define a robust, accurate and, at the same time, low-cost methodology to investigate these complexes, towards an understanding of the main factors determining the host–guest interaction and the efficacy of the drug-delivery system.

## 2. Results and Discussion

### 2.1. Melatonin: From the Isolated Molecule to the Crystal Structure

**Scheme 1:**
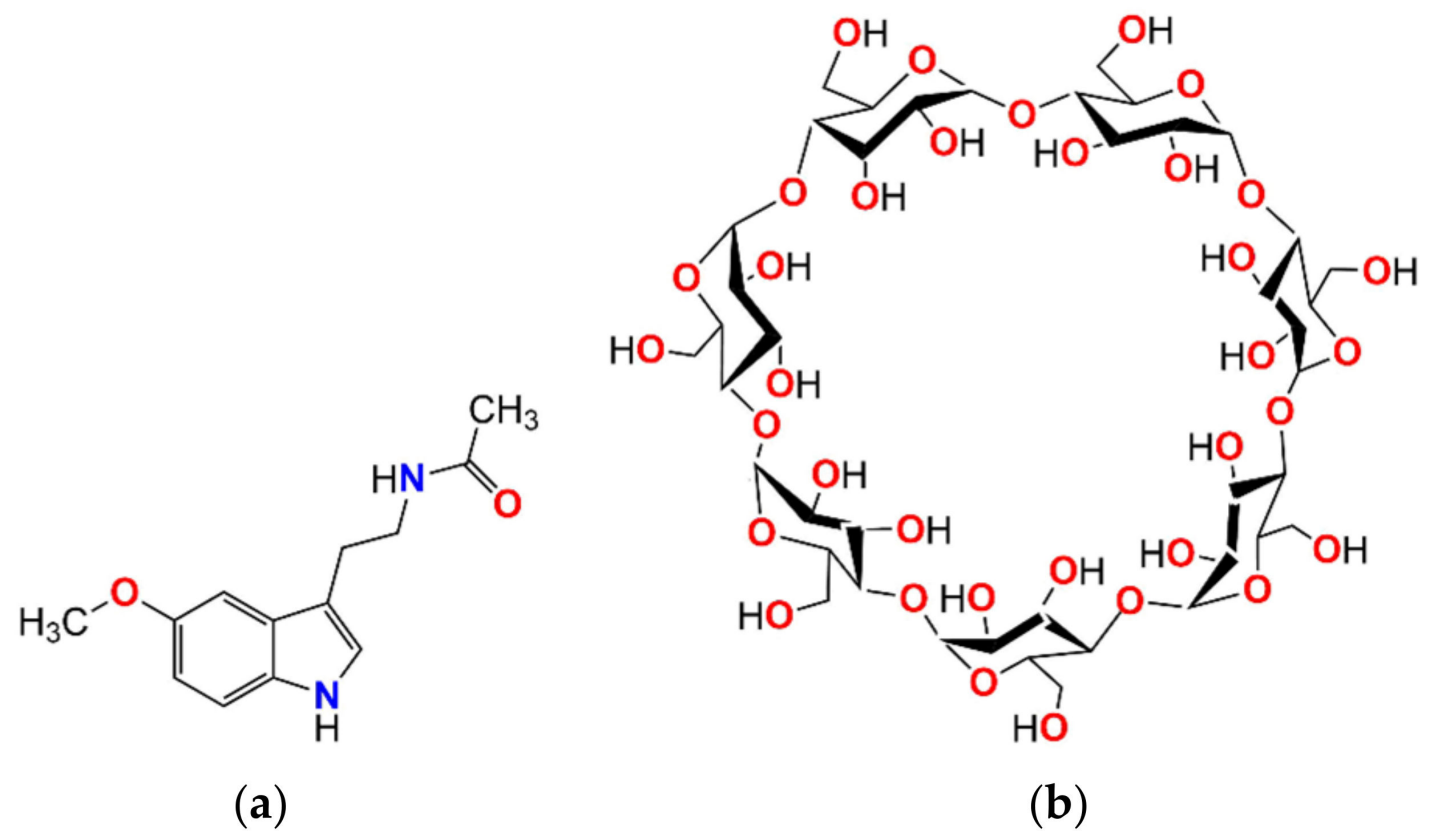
Schematic 2D representation of (**a**) melatonin, and (**b**) β-cyclodextrin.

**Figure 1:**
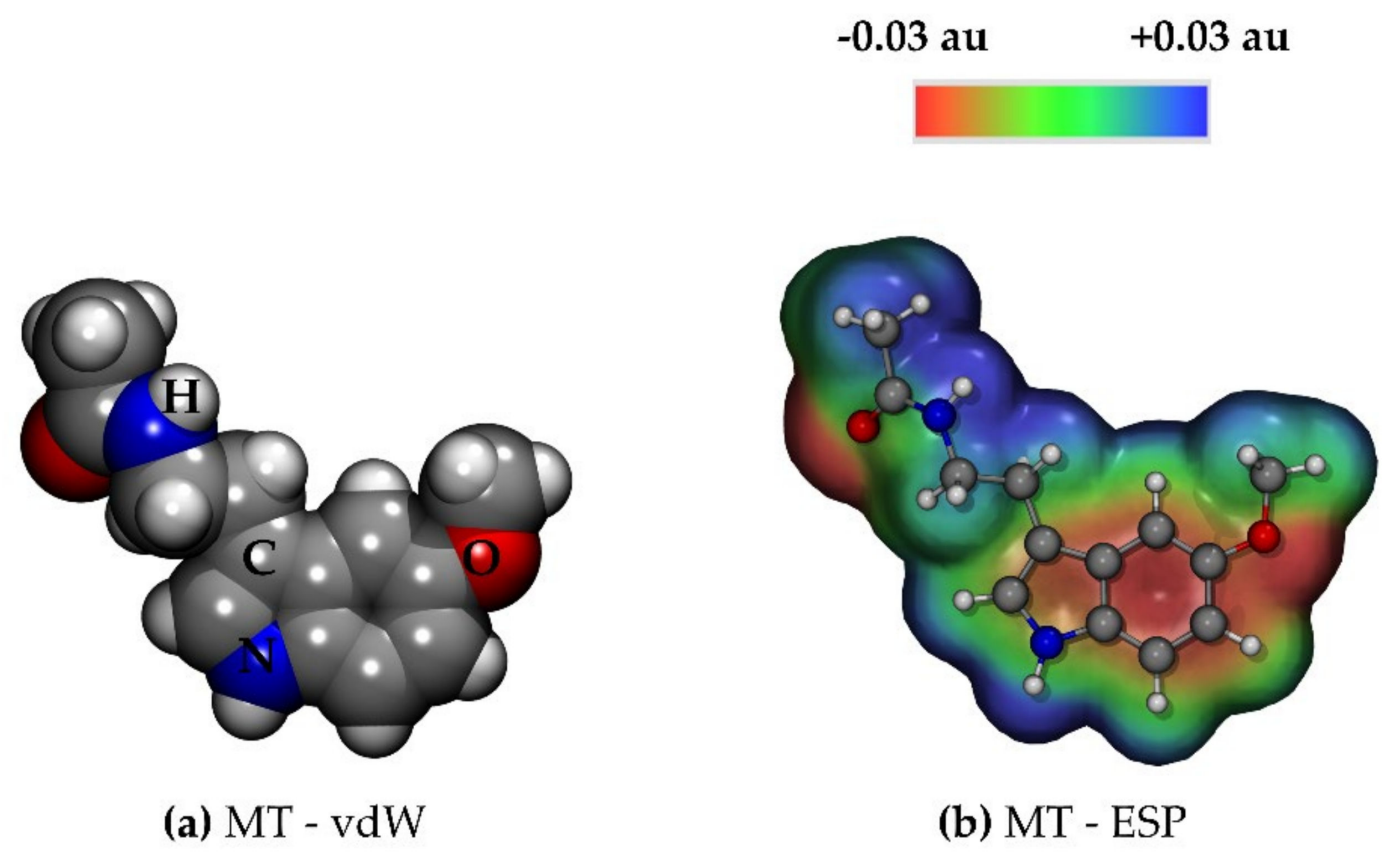
(**a**) PBEh-3c optimized structure of the extended conformer of melatonin in vdW representation. (**b**) Ball and stick representation overlapped to the ElectroStatic Potential (ESP) map plotted on the electron density (ISO electron density value = 10^−3^ au). Atom colors: H white, C grey, N blue, O red.

The structure of melatonin (MT, see Scheme 1a and Figure 1) can be divided into two parts: the indole aromatic ring (Ar) and the aliphatic chain. The former is planar and the methoxy group is oriented in the same plane of the ring due to mesomeric effects (the Ar-O presents a partial character of double bond), while the chain, thanks to the double -CH_2_- group, affords mobility and variety on the conformational space of the molecule. Intermolecular interactions among melatonin molecules are dominated by hydrogen bonds: carbonyl and methoxy groups act as hydrogen bond acceptors (red zones of the electrostatic potential map of Figure 1b), while the N-H indole and amide groups are hydrogen bond donors (blue zones of Figure 1b). Other possible interactions concern the aromatic rings, which interact with each other via van der Waals forces (π-π interactions among ring planes, red zone on the top of the indole ring). Intramolecular hydrogen bonds are not possible because the aliphatic chain is too short to allow for this kind of interaction. A search for the lowest energy minimum of this molecule was performed and benchmarked with different methods.

#### 2.1.1. Melatonin: Conformational Analysis

**Figure 2:**
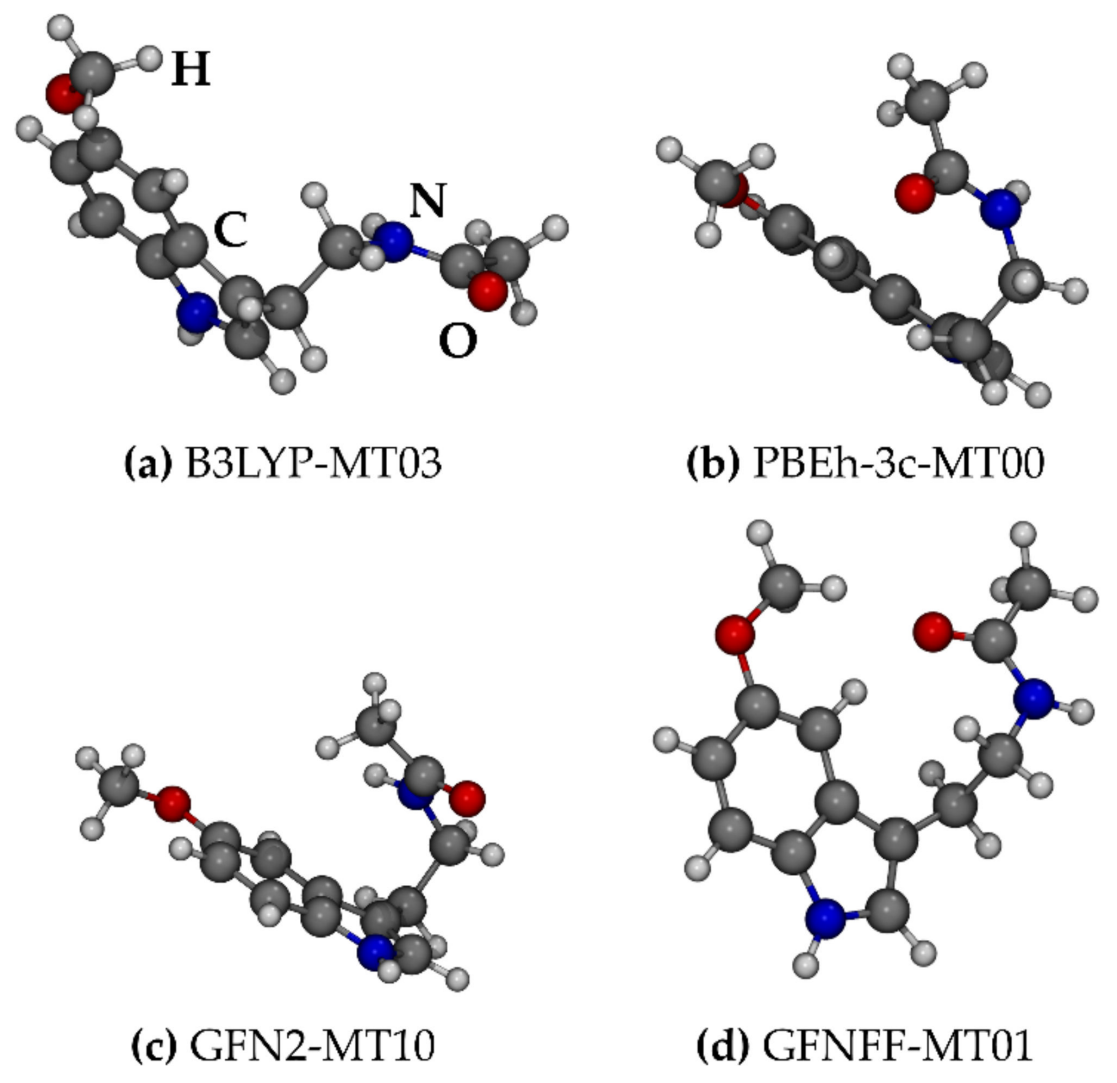
(**a**–**d**) Optimized low-energy minima of isolated gas phase melatonin (MT). In the notation MTXX, the number XX corresponds to the structure number according to the CCSD(T) ranking calculated by Fogueri et al. [47]. Atom colors: H white, C grey, N blue, O red.

With the aim of finding the lowest energy minimum of melatonin, we started from the results by Fogueri at al. [47], where the conformational space of melatonin was investigated at the CCSD(T)/cc-pVTZ level. The 52 stable conformers were re-optimized at the xTB-GFN2 level and compared with the reference (see Table S1, Supplementary Materials). Fifteen out of fifty-two xTB-GFN2 conformers (within 2 kcal/mol of relative energy with respect to the most stable structure) were reoptimized with the methodologies cited in the Methods section, i.e., B3LYP-D3BJ(ABC)-gCP (hereafter called B3LYP), PBEh-3c and xTB-GFNFF. There are some “outliers” in Table 1, namely MT18, MT21 and MT22, i.e., some structures which, according to CCSD(T) calculations, are higher in energy with respect to the 15 selected structures. Therefore, some intermediate structures are missing: MT08, MT12, MT13 and MT14. Every method provided a different lowest energy minimum of melatonin, as shown in Figure 2, with different relative energies, highlighting the general complexity of finding the low-energy conformational minimum also for relatively small molecules.

B3LYP is the only functional which finds an extended structure as a most stable conformer (see Figure 2a), i.e., with the chain which does not point towards the indole ring. For the PBEh-3c and GFN2 (Figure 2b,c) cases, the linear chain folds on the top of the indole ring, thus maximizing dispersive intramolecular interactions and the difference between the two minima concerning the relative position of CO and NH groups with respect to the indole ring. It is worth mentioning that PBEh-3c is the only functional capable of reproducing the lowest energy minimum found by Fogueri et al. [47], with the carbonyl moiety pointing directly towards the ring, while with GFN2, its place is taken by the amidic -NH. GFNFF (Figure 2d) provided a more planar structure, where one of the indole -CH- interacts with the CO with a weak hydrogen bond (-CH∙∙∙OC-). In Table 1, the relative energies for each structure compared to the lowest energy minimum of the corresponding method are listed. The window of energetic range changes considerably from one method to another. GFN2 is the method which presents the smallest energetic span (such as that at the CCSD(T) level, despite that the structure ordering is different), resulting in a 1.53 kcal/mol difference between the most and the least stable structures, the majority of which is almost energetically indistinguishable (the first 8 structures are within 0.17 kcal/mol). PBEh-3c is the functional showing the best accordance with the CCSD(T) energy ranking, and it expands the energetic range with respect to GFN2. DFT-B3LYP and GFNFF are the methods which have the best agreement with one another, both in terms of structural and energetic ranking. These results show that the exploration of the conformational space of a relatively small organic molecule, even if it does not present a large number of degrees of freedom, is a very challenging matter, also for DFT methods which are usually considered able to provide accurate results in terms of structure and energetics for these kinds of systems.

#### 2.1.2. Melatonin: Dimer

**Figure 3:**
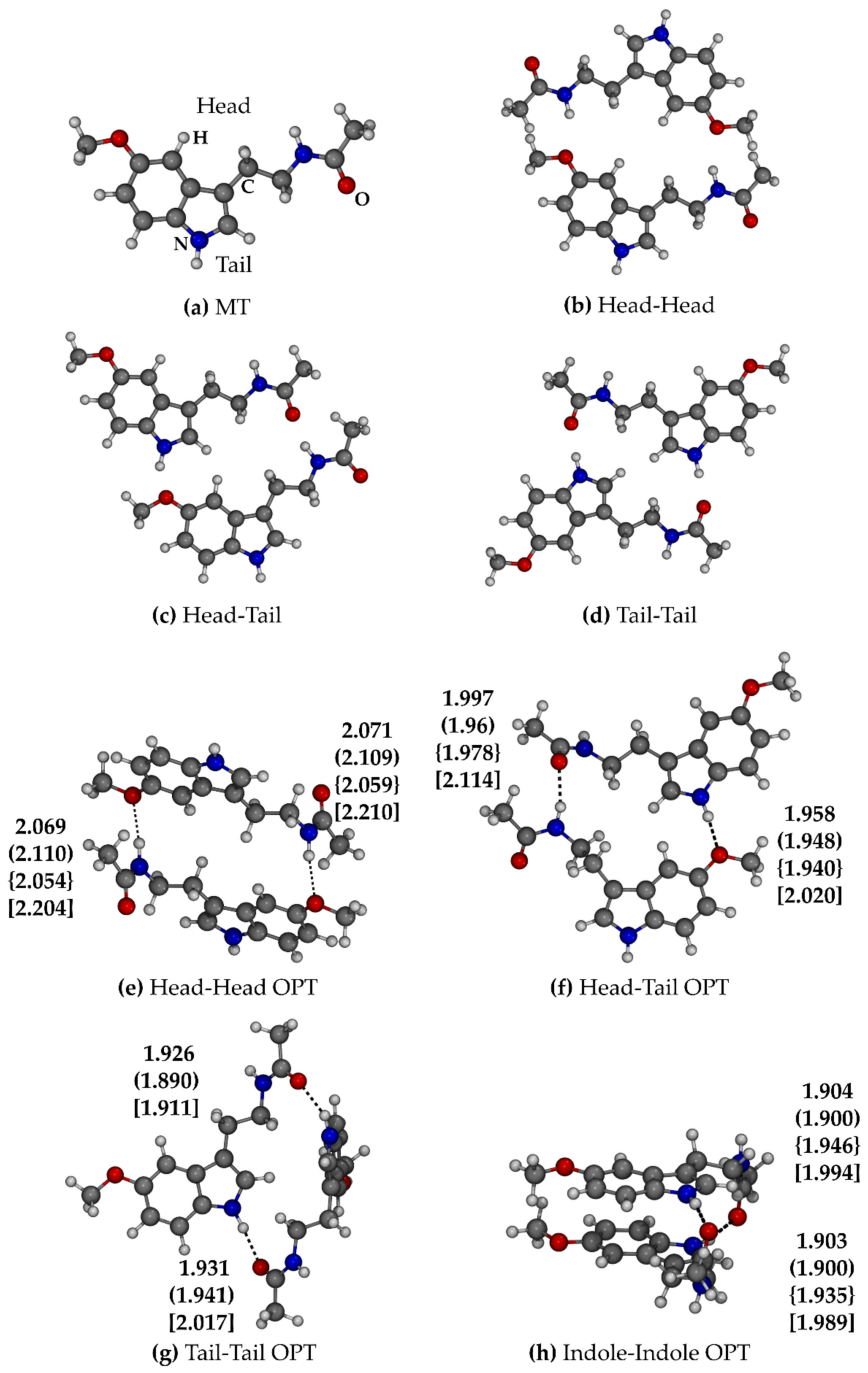
(**a**) Schematic representation of the two sides (Head and Tail) of the molecule with acceptor/donor groups. (**b**–**d**) Starting structures of melatonin dimers. (**e**–**h**) Optimized (OPT) structures of melatonin dimers. Bond length notation: GFN2, (GFNFF), {B3LYP}, [PBEh-3c]. Bond distances are in Å. Atom colors: H white, C grey, N blue, O red.

As melatonin presents two hydrogen bond donors and acceptors, its dimer (DIM) was built in three different orientations, starting from the linear extended structure: head–head (Figure 3b), tail–tail (Figure 3d) (the two melatonin molecules are in an antiparallel conformation with respect to each other) and head–tail (Figure 3c) (melatonin molecules in parallel conformation), with the aim of investigating the possible interactions between two molecules of melatonin, maximizing all the possible hydrogen bonds (the starting and optimized structures are shown in Figure 3). The major features for these cases are (see Figure S1, Supplementary Materials for a comparison of the structure obtained with each method):

- Head–Head (HH): Both the amidic N-H groups of the two melatonin molecules donate a hydrogen bond to the methoxy groups. The hydrogen bonds calculated by PBEh-3c are weaker (2.20–2.21 Å), and, accordingly, the interaction energy is lower (−2.0 kcal/mol, see Table 2) with respect to the other methods (2.05–2.11 Å, −4.0 < E < −7.5 kcal/mol of interaction energy).
- Head–Tail (HT): In this case, the indole -NH- donates a hydrogen bond to the methoxy group, while the amidic -NH- to the amidic -CO- (see Figure 3f). In this case, the structural (1.94–2.02 Å and 1.97–2.11 Å are the ranges of the two hydrogen bonds) and energetic accordance (−3.9 < E < −8.6 kcal/mol) among the methods is good. B3LYP shortens the hydrogen bonds and presents the highest interaction energy (−8.6 kcal/mol).
- Tail–Tail (TT): The hydrogen bond donors are the indole -NH- groups, while the acceptors are the amidic -CO- (see Figure 3g). The steric hindrance of -CH- and -CH_2_- groups led to a conformation with the two planes of the indoles in perpendicular orientation with one another. In this way, the two melatonin molecules can maximize their interactions; indeed, the hydrogen bonds are shorter (1.89–2.02 Å) with respect the previous cases, and the TT adduct is more stable with respect to both HH and HT (−9.0 < E < −11.3 kcal/mol). B3LYP is the only method which does not find a minimum in the structure described above, leading the two indole rings to interact with each other (Indole–Indole) through the π-electrons of the aromatic rings (π-π interaction), keeping the -NH∙∙∙OC- hydrogen bonds (see Figure 3h).
- Indole–Indole (II) was then reoptimized with the other methods for consistency. For all the methods, the hydrogen bonds are the shortest (1.90–1.99 Å); moreover, the π-π interaction leads to an extra-stabilization which makes this dimer the most stable one (−11.9 < E < −18.8 kcal/mol).

#### 2.1.3. Melatonin: Crystal

**Figure 4:**
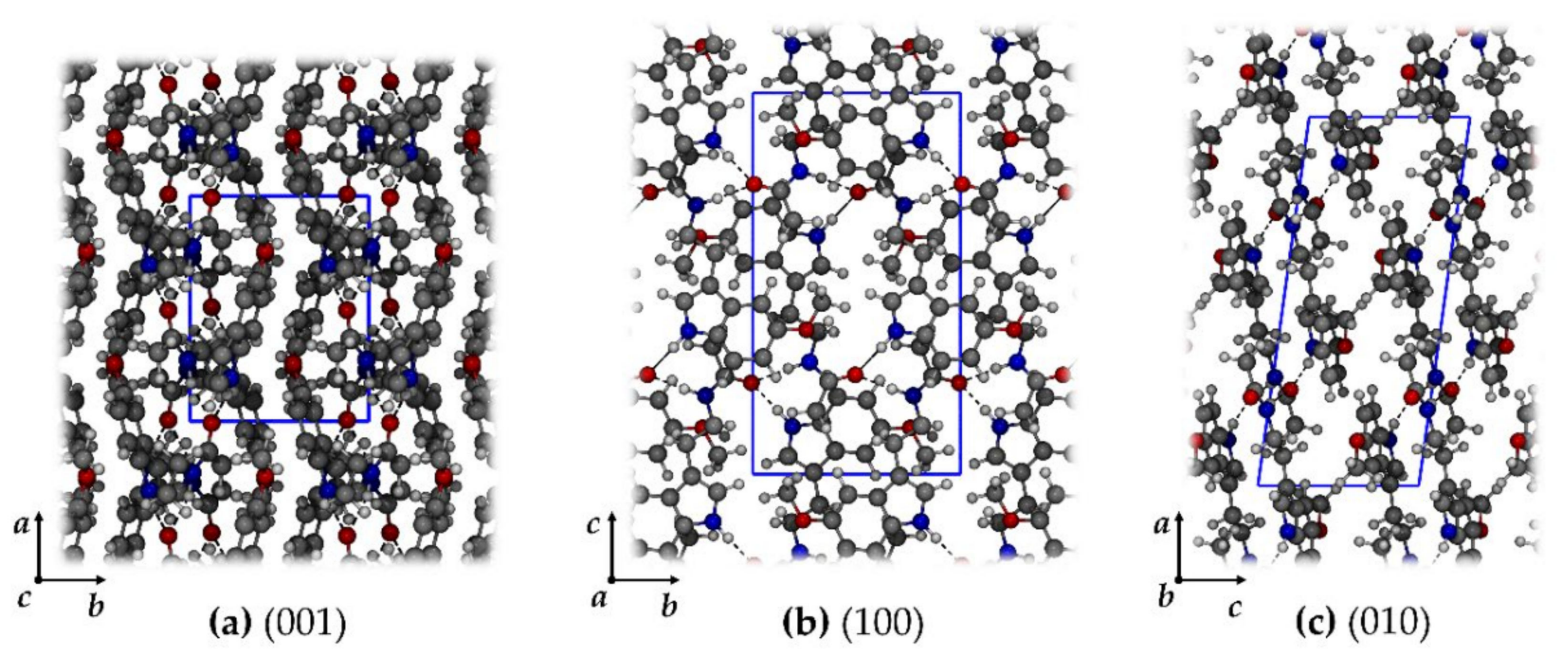
(**a**–**c**) View from (001), (100), (010) crystallographic plane respectively. PBEh-3c optimized structure of the melatonin crystal. Bond distances are in Å. Atom colors: H white, C grey, N blue, O red. Unit cell in blue.

The crystal structure of melatonin belongs to the P2_1_/c space group. The lattice is monoclinic (a = 7.714 (7.429) Å, b = 9.276 (9.286) Å, c = 17.118 (17.208) Å, α = β = 90°, γ = 96.91° (97.93), the numbers in parenthesis are the PBEh-3c optimized ones) and its PBEh-3c optimized structure is shown in Figure 4. Along the (100) and (010) crystallographic planes (Figure 4b,c), a number of hydrogen bonds are established with amidic and indole -NH- groups as donors and the CO as a simultaneous double acceptor. In the (001) plane, in contrast, only van der Walls interactions between the indole rings are established, creating a “sandwich-like” structure (Figure 4a). The calculated cohesive energy is −33.5 kcal/mol (ΔH = −32.1 kcal/mol and ΔG = −14.5 kcal/mol), which is about three times the dimerization energy of DIM-II (ΔE = −11.9 kcal/mol).

#### 2.1.4. FTIR Spectroscopy

**Figure 5:**
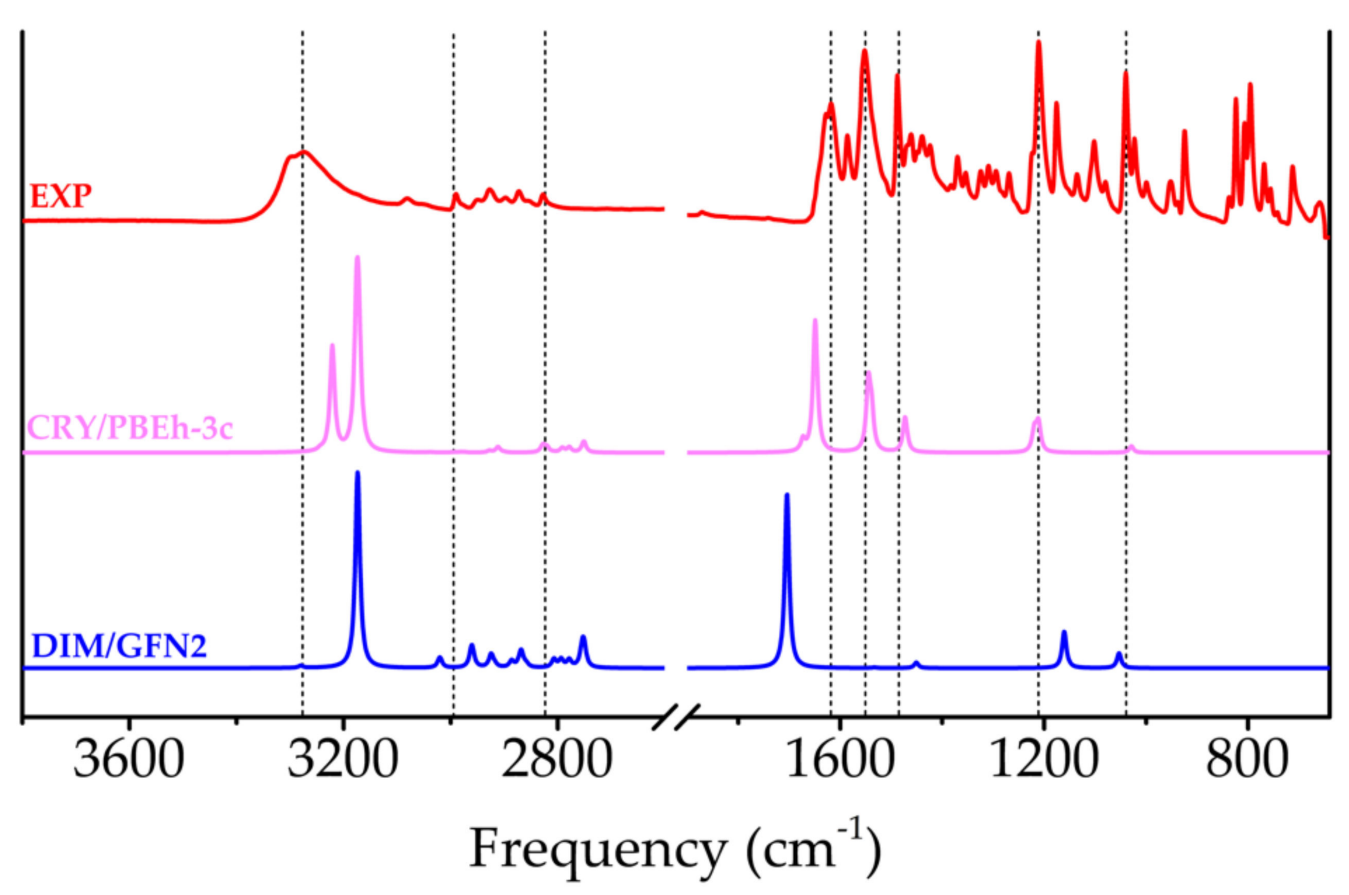
Experimental (red line) vs. computed melatonin IR spectra with anharmonic corrections. CRY/PBEh-3c corresponds to the calculated spectrum of the crystal structure at the PBEh-3c level (pink line), while DIM/GFN2 to the spectrum of the Indole–Indole dimer at the GFN2 level (blue line).

Harmonic frequencies and relative intensities for melatonin, its dimer and its crystal were computed and compared with the experimental spectrum of the solid phase. In Figure 5, the spectra presenting the best agreement with the experiment are shown: the crystal structure was simulated at the PBEh-3c level of theory (CRY/PBEh-3c), while the dimer with GFN2 (DIM/GFN2). The spectrum of the dimer was also calculated with PBEh-3c; however, the NH bands are fairly blue-shifted with respect to the experiment (see Supplementary Figure S2), at variance with GFN2, which locate the -NH- bands at the same wavenumbers as CRY/PBEh-3c. Moreover, an anharmonic correction was included to the IR spectra of Figure 5. Due to the high computational cost of this correction, it was performed on the melatonin molecule, and then the anharmonic shift calculated on the molecule was applied to the frequencies of the crystal structure. Of course, the anharmonic correction will be different for H-bearing moieties whether they are involved in possible hydrogen bonds. In our specific case, as one can see in Figure 5, the NH and CH stretching region is slightly red-shifted upon anharmonic correction. The frequency zone between 1000 and 1700 cm^−1^ is very well-represented, with a root mean square deviation of 16 and 49 cm^−1^ for CRY/PBEh-3c and DIM/GFN2, respectively.

The experimental broad band relative to the N-H stretching (amide and indole) is found around 3280 cm^−1^, while the computed spectra shift towards lower frequencies by about 100 cm^−1^. This zone is influenced by the effect of anharmonic vibrations, particularly important for bonds involving hydrogen atoms. Without corrections, the same peaks would be located at 200 cm^−1^ higher frequencies, highlighting the anharmonic effect. Between 3000 and 2800 cm^−1^, the vibrational modes regarding C-H stretching, both aromatic and aliphatic, are located. Including the anharmonicity corrections, the positions and the shapes of the peaks show an almost perfect match with the experiment.

The CO moiety is particularly important in FTIR spectra, due to the fact that it is usually in a clean zone of the spectrum and has high intensity, and accordingly, it is easily identifiable; experimentally, it appears at 1617 cm^−1^, with CRY/PBEh-3c at 1649 cm^−1^ and with DIM/GFN2 at 1706 cm^−1^, thus confirming the increasing agreement of the computed spectrum going from the molecular case to the crystal, which is a more realistic model of the system. Stretching of aromatic C=C double bonds also shows good accordance with experimental results: the peak is located at 1551 cm^−1^ and the computed shifts at 1545 cm^−1^ for CRY/PBEh-3c and at 1539 cm^−1^ for DIM/GFN2 (very low intensity). At 1488 cm^−1^, the bending frequencies of the aliphatic C-H group are located, and the shifts with respect to the computed spectrum are low for both PBEh-3c/CRY (1474 cm^−1^) and DIM/GFN2 (1452 cm^−1^). The peak of the Ar-O bond appears at 1210 cm^−1^ in the experimental plot, showing a perfect agreement with CRY/PBEh-3c (1210 cm^−1^) and a big shift with DIM/GFN2 (1162 cm^−1^), also due to the corrections applied. This bond has a percentage of double bond character, which leads to planarity (-OCH_3_ is always on the same plane of the indole ring). Lastly, good accordance is found for the O-CH_3_ stretching, placed at 1040 cm^−1^ in the experiment, at 1032 cm^−1^ in the computed CRY/PBEh-3c spectrum and at 1057 cm^−1^ for DIM/GFN2. The normal symmetric and antisymmetric modes of these last two signals are located a few cm^−1^ from the frequencies described above.

### 2.2. β-Cyclodextrin

**Figure 6:**
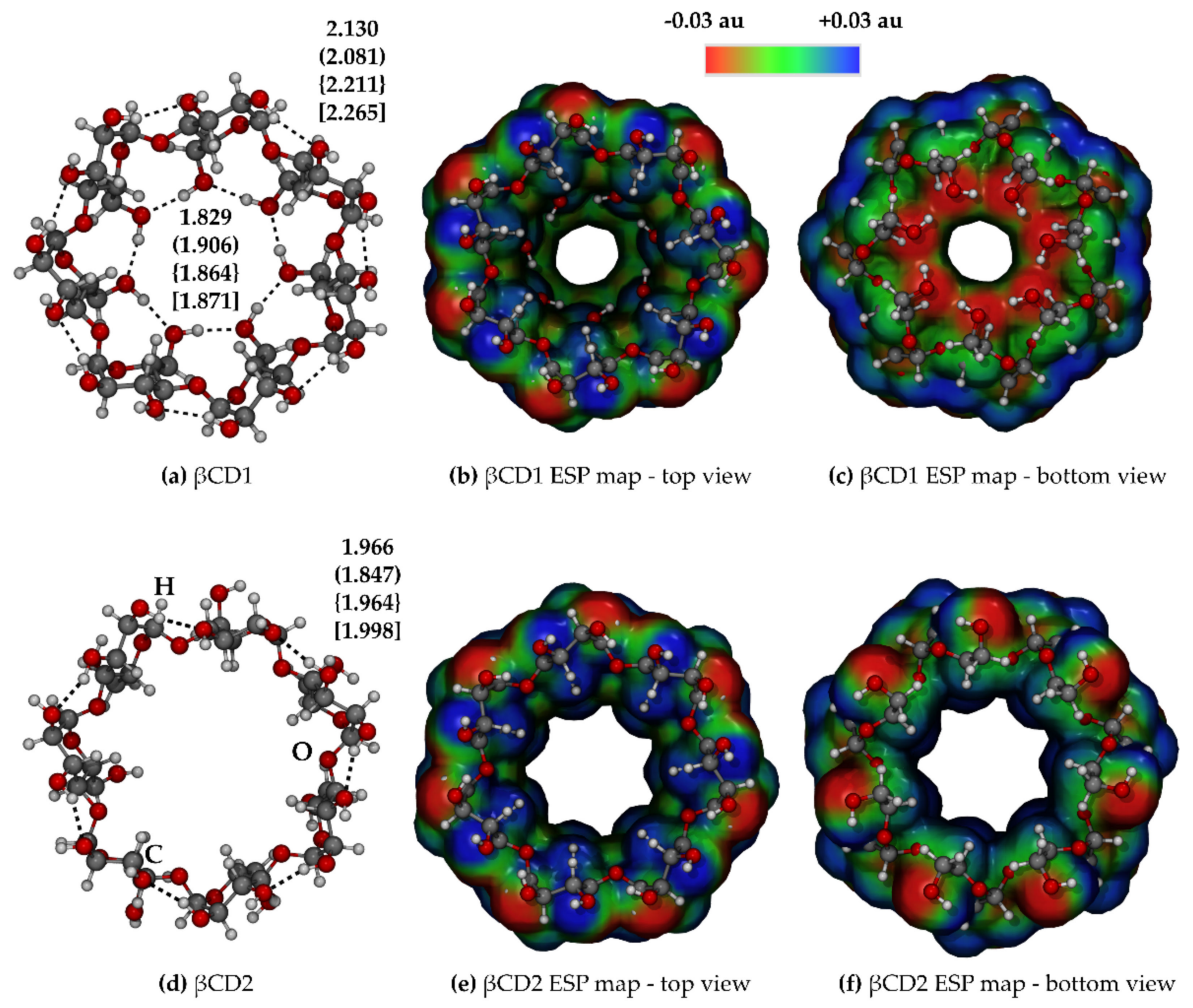
(**a**) Optimized structure of β-cyclodextrin. Bond length notation: GFN2, (GFNFF), {B3LYP}, [PBEh-3c]. (**b**,**c**) β-cyclodextrin structure superimposed on the ElectroStatic Potential (ESP) maps plotted on the electron density (ISO electron density value = 10^−3^ au). Bond distances are in Å. Atom colors: H white, C grey, N blue, O red.

β-cyclodextrin (βCD) has a cyclic structure made by seven units of D-glucose bonded with a glycosidic bridge (Scheme 1b), providing the covalent skeleton to the molecule. Each monosaccharide has three hydroxyl groups: two of them, on C_2_ and C_3_, are located on the outermost top part of the βCD ring, while the -OH group on C_6_ in the outermost bottom part has high flexibility due to the free rotation on C_6_ (see Supplementary Figure S3). In this case, the hydrogen bonds that can be established between -OH groups have a crucial role in stabilizing the molecule, with a symmetric pattern of intramolecular forces that affords regularity and structural stability to the ring. A single molecule of βCD can form up to 14 internal hydrogen bonds, 7 with the C_2_ hydroxyl group behaving as a hydrogen bond donor and the C_3_ -OH on the following glucose as an acceptor, and the other 7 among the hydroxymetil group on C_6_, depending on the conformer. The other hydroxyl <<<<group on C_3_ is involved in hydrogen bonds (both as donors and acceptors) in the crystal structure of βCD. The two isomers analyzed in the present work (namely CD1 and CD2, see Figure 6) were taken from [48], where a deeper analysis of the possible CD conformers is available. The two conformers have the difference that in CD1, the hydrometil group on C6 atoms interact among each other, thus reducing the hole diameter from 6.4 to 9.6 in CD2. Results in Table 3 show that there is a good agreement between results at B3LYP and GFN2 levels (CD1 more stable by about 7 kcal/mol), however, at PBEh-3c level CD2 conformer is more stable than CD1 by 4.4 kcal/mol. These results show that the conformational exploration of a relatively simple organic molecule has to be treated carefully.

Moreover, we also optimized both the conformers in implicit solvation at GFN2 and GFNFF levels. In all the cases, the most stable structure results to be CD2 (see Figure S4, Supplementary Materials for a comparison of the structure obtained with each method). According to these results, we used CD2 as a host structure for the melatonin molecule, as the experiments were carried out in water solution. 

Figure 6 shows the electrostatic potential maps (ESP) of the two βCD conformers studied in this work, highlighting the cavity in the center of βCD, where small and medium-size organic molecules can be placed. The majority of negative potential (red zone) is found in correspondence to the hydroxyl groups, and they indeed act as strong hydrogen bond donors and acceptors; in contrast, the glycosidic oxygens are shielded by aliphatic groups, even if they can accept one hydrogen bond (small green zones inside the ring). These features are particularly important to provide a proper starting orientation to the guest molecule, thus maximizing all the stabilizing interactions. 

### 2.3. Inclusion Complex

The inclusion complex between melatonin and βCD was formed with the manual insertion of a guest molecule into the cavity, using the molecular modeling software MOLDRAW [49], with the aim of maximizing the electrostatic host–guest interactions (both having donor and acceptor hydrogen bond groups). As a first case, to compare the results obtained with xTB and DFT methods, the extended structure of melatonin was inserted inside the βCD and the geometry was optimized. Further inclusion complexes were then considered employing only the xTB-GFN method. In addition to the evaluation of the complexation energy, thermodynamic corrections were also calculated and discussed. FTIR computed spectra were used as a tool to validate the approach with the novel xTB method. The inclusion of implicit solvation effects was probed by performing molecular dynamics simulations in both gas and liquid (water and acetonitrile) phases on the most stable complex, with the aim of studying the inclusion and evolution of the MT/βCD complex, and the melatonin wash-out process.

#### 2.3.1. Extended Melatonin Inclusion Complex: Method Benchmark

**Figure 7:**
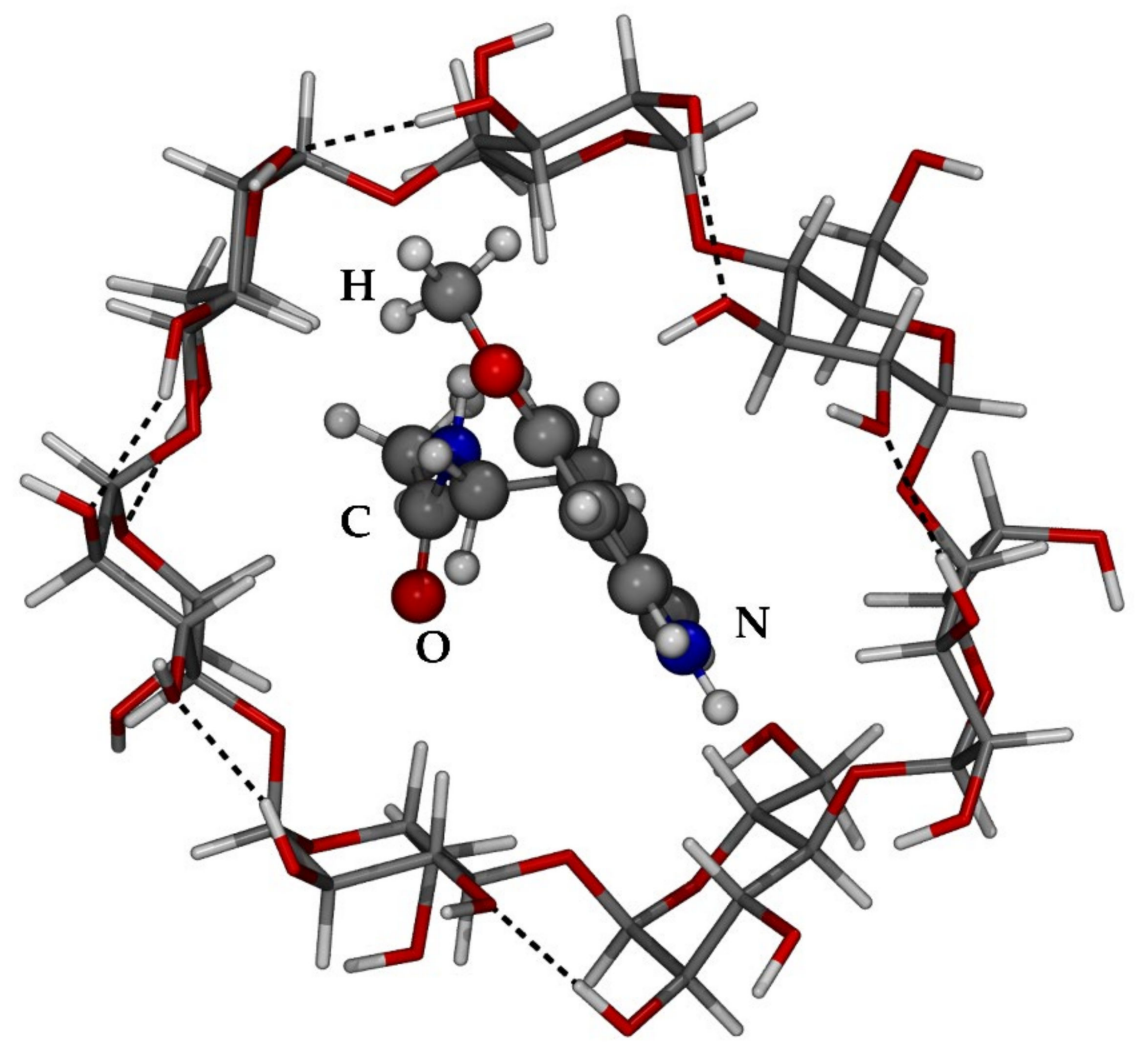
xTB-GFN2 optimized structure of the inclusion complex of extended melatonin into β-cyclodextrin.

The complex with extended melatonin was studied both with DFT and xTB methods, in order to make a comparison in terms of complexation energy. The complexation energies have been obtained with a single-point calculation with all the previously described methodologies on the GFN2 optimized geometry (Figure 7). In this way, we can determine how the different methodologies evaluate the interactions of the MT/βCD complex at fixed geometry. In Table 4, the complexation energies are shown. Values show a good agreement among the methods, considering the large differences both in terms of accuracy and computational cost. GFN2 reports the lowest complexation energy (ΔE_disp_ = −30.3 kcal/mol), while GFNFF overestimates this interaction (ΔE_disp_ = −41.3 kcal/mol). DFT-based methods stay in between, obtaining very similar results (ΔE_disp_ = −34.5 kcal/mol for PBEh-3c and ΔE_disp_ = −35.3 kcal/mol for B3LYP). Moreover, we also separated the electrostatic from the dispersive contributions, in order to understand the main forces responsible for the MT/βCD interaction. Of course, each method includes by definition a certain amount of dispersion, and therefore, the dispersive contribution will be different from one method to another. From Table 4, it can be seen that a large part of the interaction is indeed driven by dispersion, thus highlighting the importance of taking into account a correct treatment of weak forces.

xTB computational performances are the major strength of this method: on an 8-core CPU, a geometry optimization and frequency calculation for the inclusion complex took about 7 min for the GFN2 level and about 1 min with GFNFF. The same calculations require approximately 9 h for PBEh-3c and 30 h for B3LYP on a 24-core CPU. Agreement between these low-cost methods is not perfect, but in most cases, the errors are acceptable, in particular considering the good results of the computed GFN2 IR spectra. Moreover, GFN2 allows to perform large-scale molecular dynamics simulations, following the evolution of this system over a timescale which would be unaffordable for DFT methods.

#### 2.3.2. Melatonin/β-Cyclodextrin Complexes

**Figure 8:**
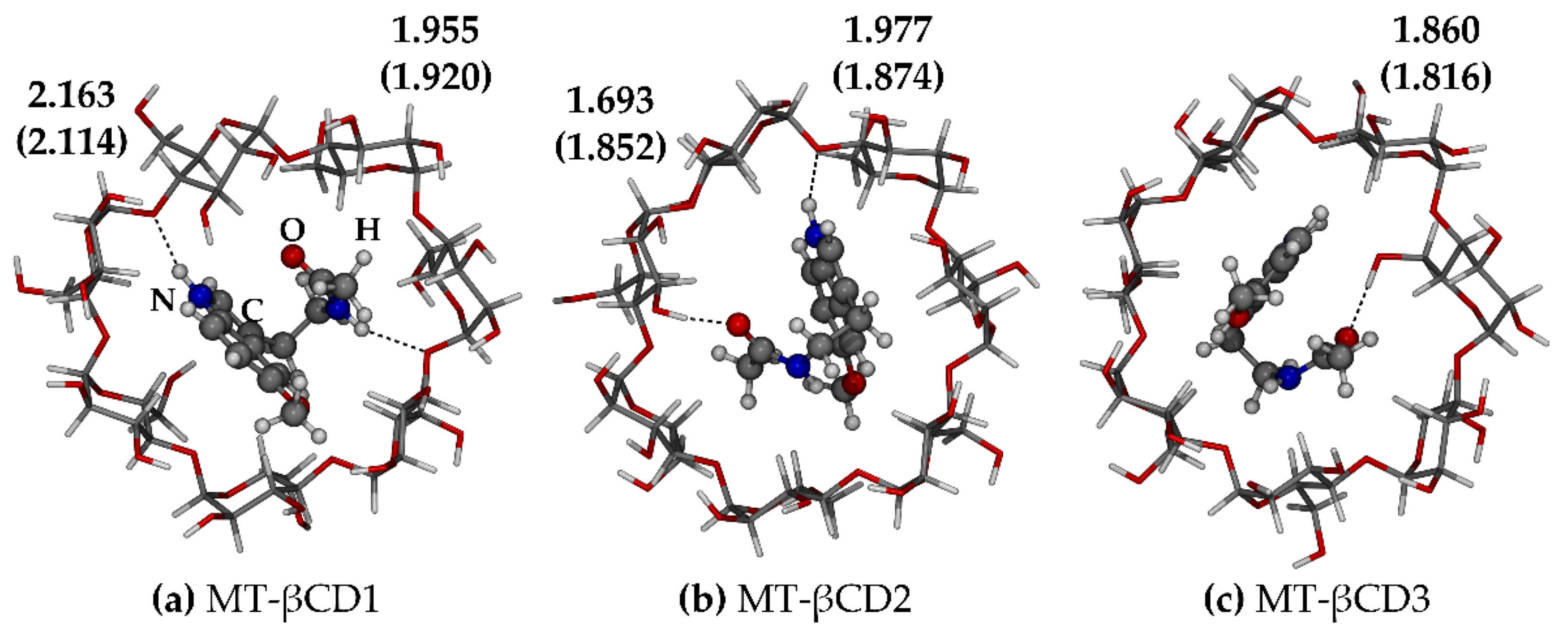
xTB-GFN2 optimized geometries of the studied MT-βCD inclusion complexes. Bond length notation: GFN2, (GFNFF). Bond distances are in Å. Atom colors: H white, C grey, N blue, O red.

With the purpose of exploring different possible intermolecular host–guest interactions, three different starting positions of the most stable melatonin conformer, found by xTB-GFN2, were built. In Table 5, the complexation energies (also including thermodynamic corrections) in gas phase and in the presence of the solvent are reported for all the cases at the GFN2 level (data at the GFNFF level are available in Table S2, Supplementary Materials), i.e., the extended (EXT) melatonin inclusion and the three studied cases (MT-βCD1, MT-βCD2, MT-βCD3). In Figure 8, the optimized structures are reported. The most stable conformer is MT-βCD2 (ΔE = −39.5 kcal/mol, Figure 8b), which presents the strongest hydrogen bonds among the three structures. As one can see from Table 5, thermal corrections (ΔH) do not substantially affect the complexation energy. The inclusion of entropic effects does not favor the complexation process (ΔG_MT-βCD2_ = −16.9 kcal/mol), which in all cases is still favored with respect to the two isolated structures. Not surprisingly, when the effect of the solvent is also considered, the free complexation energy becomes positive in all cases, and this explains the usage of water and acetonitrile as solvents to wash-out the melatonin from the inclusion complex.

GFNFF tends to overestimate the energies compared with GFN2, except for MT-βCD2, whose complexation energy results very similar to that calculated at the GFN2 level (ΔE = −40.6 kcal/mol). Curiously, according to GFNFF results, these three structures are almost indistinguishable from an energetic point of view, with the values of complexation energy varying from −39.4 to −41.2 kcal/mol. 

#### 2.3.3. FTIR Spectroscopy

Harmonic frequencies and relative intensities for the most stable inclusion complex (MT-βCD2) were calculated, and the IR spectrum was plotted and compared with the experimental hyper-crosslinked inclusion complex of melatonin and β-cyclodextrin in the form of a nano-sponge, obtained in our laboratories.

**Figure 9:**
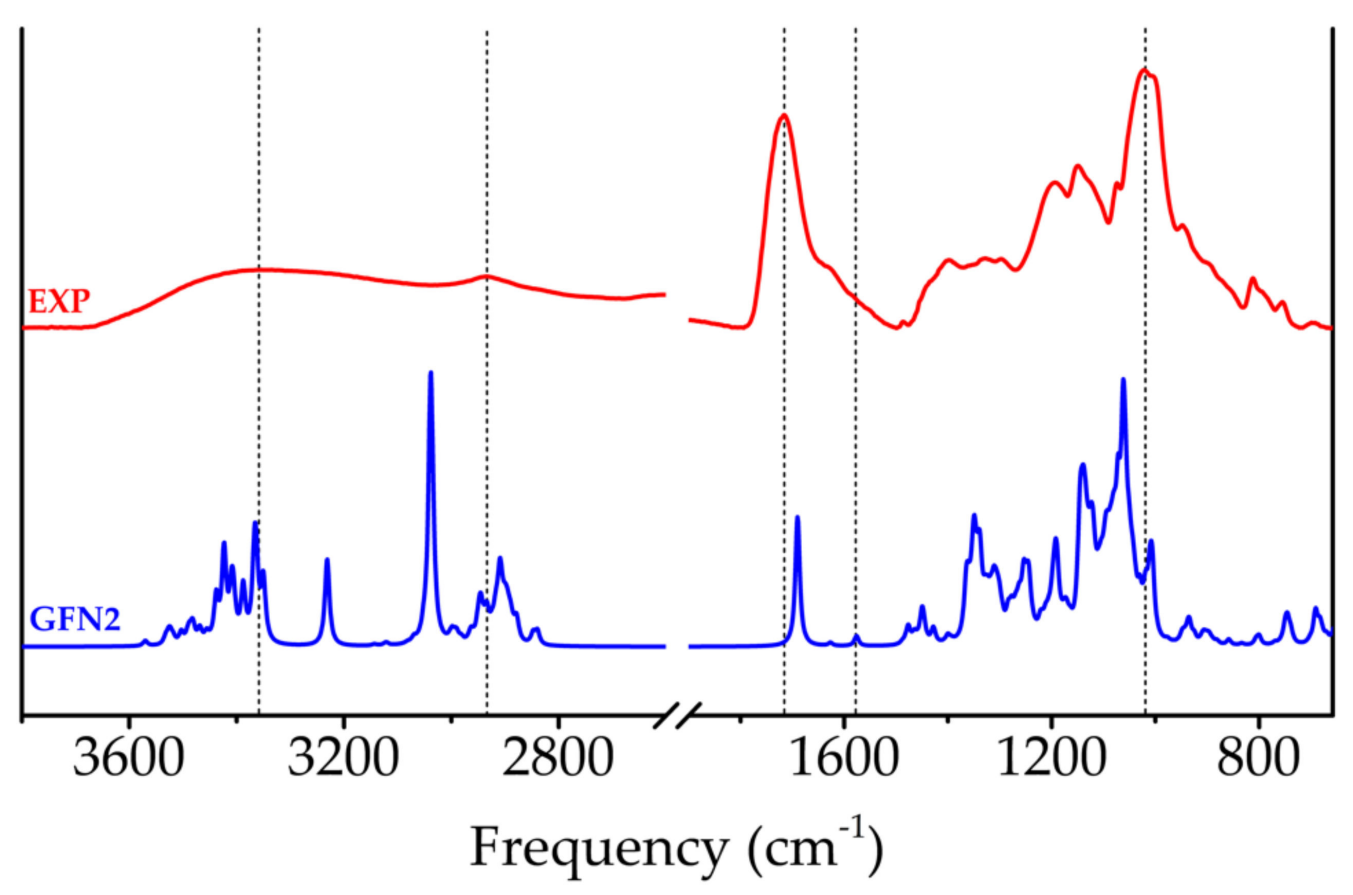
Experimental (red line) vs. GFN2-computed (blue line) spectra of the most stable inclusion complex (MT-βCD2).

The computed IR spectrum of Figure 9 can be used as a yardstick to evaluate the quality of results and the reliability of computational methods used. Owing to the high calculation cost of frequencies, this was only possible with xTB methods.

The very broad band at about 3300 cm^−1^ is relative to all the possible N-H and O-H stretching modes: the former is present only in melatonin molecules while the latter is found in cyclodextrin. The computed GFN2 spectrum finds a series of peaks with different intensity, centered approximately in the same zone as the experiment. The experimental peak related to C-H stretching (aliphatic and aromatic) looks like a broad band in the range 3000–2800 cm^−1^, with a very low intensity. Once again, the GFN2 peak is located almost at the same frequencies. However, between the OH/NH and CH regions, there are two intense lonely peaks related to two hydroxyl groups which are involved in very strong hydrogen bonds. Stretching C=O is present with an intense peak at 1708 cm^−1^ in the experimental spectrum, and at 1726 cm^−1^ at the GFN2 level. Stretching of aromatic C=C double bonds is present in computed spectra, differently from the experiment where these signals, typical of isolated melatonin, are probably covered by the larger amount of cyclodextrin C-H and O-H bending and by C-O stretching modes. The fingerprint area is dominated by wide and quite intense peaks, especially in the range between 1500 and 1000 cm^−1^: they are combined modes referring to C-O and C-C vibrations of the cyclodextrin glucose rings. GFN2 shows a blue-shift of about 60 cm^−1^ with respect to the experiment. Despite the complexity of the system, in general, there is a good agreement between calculated and experimental spectra, thus confirming the excellent trade-off between the accuracy and the computational cost of GFN2.

#### 2.3.4. Molecular Dynamics Simulations

Thanks to the low computational cost and to the accuracy of results obtained with the GFN2-xTB method, molecular dynamics simulations were performed in gas phase (GP) to observe the evolution of the inclusion complex over time, and in the two solvents (water (W) and acetonitrile (ACN)) to study the wash-out process.

**Figure 10:**
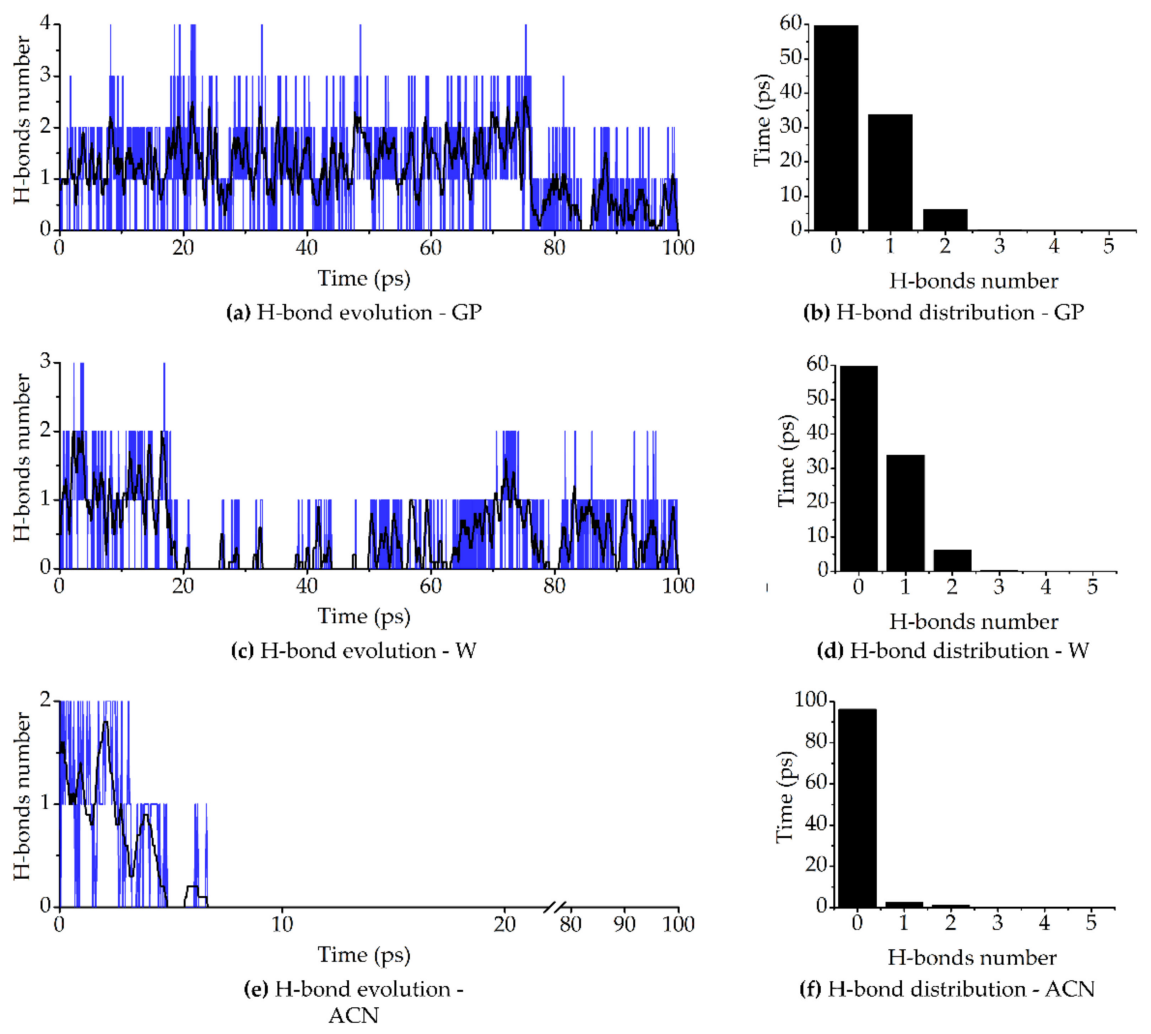
Left panels: Moving average (black line) and single values (blue line) of the hydrogen bonds’ evolution in the inclusion complex MT-βCD2. Right panels: Hydrogen bond distribution during the MD. H-bond threshold parameters: distance cutoff 3.2 Å, angle cutoff 180 ± 25°.

In Figure 10, the evolution of hydrogen bonds and their moving average of 0.5 ps are shown (left part), while the histograms report the hydrogen bond distribution over the entire MD (right part). The average number of hydrogen bonds mainly oscillates between 1 and 2 in gas phase (Figure 10a), confirming that melatonin usually remains connected to cyclodextrin by at least one H-bond. At approximately 75 ps, the number of hydrogen bonds decreases to 0–1, maximizing the dispersive forces, but always staying in the cavity. The count of hydrogen bonds points out that for much of the simulation (40 ps), one hydrogen bond is present, and for 29 ps, two hydrogen bonds, thus covering the majority of the simulation time. Only for 25 ps were no hydrogen bonds established between melatonin and cyclodextrin. Three H-bonds were reached for 5.3 ps and even four intermolecular interactions for 0.5 ps, involving in this case all the possible donor and acceptor groups present in the melatonin molecule (more details in Supplementary Figure S5).

It is interesting to observe what happens when implicit water and acetonitrile are included in the simulation (Figure 10c–f). As one can see, in water, melatonin tends to exit from cyclodextrin at about 20 ps (Figure 10c), losing some of the hydrogen bonds, even if it still interacts with the cyclodextrin, at least with dispersive forces. In acetonitrile, within 10 ps of MD, melatonin loses all the interactions with cyclodextrin, and does not go back as in water, getting farther and farther from cyclodextrin (see Supplementary Figures S6 and S7). These results are in line with the statically computed free energies of complexation, which are positive for the complex in the presence of the solvent, and also with the experimental wash-out setup.

## 3. Materials and Methods

### 3.1. Computational Details

The initial structure of linear melatonin was obtained from the Crystallographic Open Database, resolved by A. Mostad and C. Romming [50], while the β-cyclodextrin crystal structure was found in the Cambridge Crystallographic Data Center [51]. Starting from the original .cif file, with the molecular modeling program MOLDRAW [49], it was possible to obtain the .xyz file, used as inputs for computational software. 

All the calculations have been performed employing different codes (xTB, ORCA, CRYSTAL and Gaussian16), in order to test the reliability of the semiempirical functional implemented in the xTB code. More details about the different computational software used will be provided in the next sections. 

#### 3.1.1. xTB

Simulations with xTB code [52,53] (v. 6.4.0) were carried out at two different levels of theory: semiempirical with xTB-GFN2 [54] and force-field with xTB-GFNFF [55]. The acronym stands for Geometry, Frequency, Noncovalent, eXtendend Tight-Binding, highlighting the accuracy of this method in providing reasonable structures, vibrational properties and a good description of weak interactions, at a reduced computational cost, as it relies on Tight-Binding methodology. More specifically, GFN2 uses a minimal atom-centered basis set and a Tight-Binding-based Hamiltonian. On the contrary, the GFNFF method describes molecules with the rules of classical mechanics, with a self-parametrized force-field. Both support the possibility of simulating solvent effects, using the Analytical Linearized Poisson-Boltzmann (ALPB) implicit solvation model [56]. Therefore, geometry optimization and molecular dynamics simulations were also simulated in water and acetonitrile. 

For all the calculations (geometry optimizations, frequency calculations and molecular dynamics), the default settings were used (energy convergence E_conv_ = 5 × 10^−6^ E_h_, gradient convergence G_conv_ = 1 × 10^−3^ E_h_·α^−1^, accuracy for integral cutoffs and SCF criteria = 1.0). Vibrational frequencies were calculated at room temperature (298 K) to include thermal corrections to enthalpy and free energy and to verify that the optimized geometries are minimum structures on the potential energy surface (check for no imaginary modes).

The dimerization and complexation energies (ΔE) were calculated as follows:

ΔE = E_complex_ − (E_MT_ + E_βCD_) (1)

where E_complex_, E_MT_ and E_βCD_ represent the relative energies of complex (or dimer), free melatonin and free βCD respectively, at their optimized geometry. In the same way as in Equation (1), the corresponding complexation enthalpy and free energy were also calculated.

MD simulations were performed using a Berendsen thermostat at 298 K in the NVT ensemble, with timesteps of 2 fs and total time of simulation of 100 ps. The H atom mass and SHAKE algorithm were fixed to the default parameters, respectively 4 amu and algorithm #2 (i.e., all the covalent bonds were fixed). The analysis of trajectories and image editing and rendering were performed with the VMD package [57].

#### 3.1.2. ORCA

The structures of melatonin, β-cyclodextrin and the corresponding inclusion complexes were optimized in gas phase with DFT-based methods, using the ORCA (v. 4.2.1) code [58]. For the DFT calculations, two different functionals were used: PBEh-3c and B3LYP. PBEh-3c belongs to the composite DFT methods, which consist of low-cost methods based on a small basis set (def2-mSVP in the specific case), with some empirical a posteriori corrections (3c), which ensure a good accuracy in structural and electronic properties: (i) the D3 [59] correction, using the Becke-Johnson (BJ) damping scheme [60], and including the Axilrod-Teller-Muto three-body term (ABC) [61,62], the geometrical counterpoise gCP [63,64] and small modifications of the basis set to account for its incompleteness in order to obtain a good match with experimental bond lengths. On the other hand, B3LYP [65] employs the well-known hybrid exchange Becke (3 parameters) and the correlation Lee-Young-Parr [66] functionals, and all the atoms were described through the Ahlrichs def2-TZVP [67] basis set, which was chosen according to its intrinsic accuracy and small basis set superposition error. The same empirical corrections developed by Grimme as for PBEh-3c were applied, i.e., D3BJ ABC and gCP. The DFT integration grid was set to Grid4 (optimization) FinalGrid5 (single-point calculation after geometry optimization). The tolerance on energy convergence of the self-consistent field (SCF) procedure was set to the default, while on gradients and displacements of the geometry optimizer, it was tightened to 0.00003 au and 0.0006 bohr, respectively. Due to the high computational cost of B3LYP/def2-TZVP, the calculation of vibrational frequencies was performed only with the PBEh-3c functional. The dimerization and complexation energies (ΔE) were calculated as in Equation (1).

#### 3.1.3. CRYSTAL

Calculations on the crystal structure of melatonin were performed with the CRYSTAL17 [68,69] periodic code, using the cost-effective PBEh-3c method to be consistent with molecular calculations. The tolerances on energy for the convergence of the SCF procedure were set to 10^−8^ and 10^−10^ for geometry optimizations and frequency calculations respectively, while on RMS gradients and displacements of the geometry optimizer, they were tightened to 0.00003 au and 0.00012 bohr, respectively. Tolerances in the integral calculation were set to 10^−7^ Hartree for Coulomb overlap, Coulomb penetration, exchange overlap and exchange pseudo-overlap in the direct space, and to 10^−25^ Hartree for the exchange pseudo-overlap in the reciprocal space. The Pack-Monkhorst/Gilat shrinking factors for the reciprocal space were set to 2. Vibrational frequencies were computed at the Γ point by numerical differentiation of analytical first derivatives (three displacements for each atom of the unit cell, one for each cartesian direction) [70,71]. Intensities were computed through the Berry Phase approach in order to simulate the IR spectrum. The cohesive energy of the crystal was calculated as:

$$\Delta E_{cohesive}=\frac{1}{4}E_{crys}-E_{mol}$$

where *E_crys_* and *E_mol_* are the total energy of the crystal and the isolated molecule, respectively. The normalization factor is due to the number of melatonin molecules in the crystal structure.

#### 3.1.4. Gaussian

The Gaussian code was used in order to evaluate the anharmonic correction to the vibrational frequencies. Due to the high cost of this calculation, the anharmonic correction was calculated only on the free melatonin molecule. Unfortunately, composite methods (such as PBEh-3c) are not available in Gaussian, and therefore, the anharmonic correction was calculated at a good level of theory, i.e., B3LYP-D3BJ/TZVP, and then applied to the IR spectrum computed by CRYSTAL at the PBEh-3c level.

### 3.2. Experimental Details

#### 3.2.1. Synthesis of Inclusion Complex

Melatonin was purchased from Sigma-Aldrich (3050 Spruce Street, Saint Louis, MO 63103, USA) and used as-received. β-cyclodextrin was donated by Roquette Italia S.p.A., Cassano Spinola (AL) and Roquette Frères, Lestrem (Francia), and dried before use with oven treatment at 100 °C. Solid MT/βCD inclusion complex was prepared in the form of a nano-sponge, similar to the β-cyclodextrin pure citric nano-sponge previously synthesized in our laboratories [72]. βCD (5 g), melatonin (0.1025 g), catalyst sodium hypophosphite monohydrate (0.9075 g) and cross-linking agent citric acid (6.75 g) were dissolved in a beaker with stirring in 40 mL of water at 50 °C on a hot plate, until the solution was clear. Next, the solution was transferred to an oven and maintained at 140 °C for 1 h and at 100 °C for 16 h. The presence of an insoluble precipitate, recovered in crystal form, indicates the completeness of the crosslinking reaction. The product was then crushed until a powder was obtained and stored at room temperature.

#### 3.2.2. FTIR Spectroscopy

FTIR spectra were recorded with a PerkinElmer Spectrum 100 with DTGS (triglycine sulfate deuterated) detector. Parameters used were: spectral window from 4000 to 650 cm^−1^, resolution 4 cm^−1^, pressure applied on sample 100–120 N and with 16 scans per spectrum.

The FTIR spectra were performed on a powder sample of melatonin, used without further purification, and on the experimental hyper-crosslinked inclusion complex between melatonin and β-cyclodextrin in the form of a nano-sponge, synthesized with the procedure described in Section 3.2.1.

## 4. Conclusions

In the present work, both MT and βCD bare structures were studied, as well as possible guest structures of inclusion complexes, by means of static and dynamic simulations using different computational methods, from molecular mechanics, to semiempirical, to ab initio. The idea was to find a cost-effective methodology with an accurate description of the intermolecular forces acting on this system. We do not claim to provide the actual structure of MT/βCD complex: the idea was to create a cheap but accurate method, capable of managing more complex and realistic systems, i.e., a structural model of a nano-sponge, comprised of several βCD units linked to each other through the molecule used as a cross-linker agent, also including micro-solvation water molecules. An accurate analysis of the different methods was performed, as well as a comparison with experimental IR spectra. Results clearly show that the xTB-GFN2 method provides very accurate results in good agreement with the experiment, in the same way as DFT methods do, but at a reduced computational cost, 2/3 orders of magnitude less than a typical DFT functional. Molecular dynamics simulations have been performed in gas phase to provide an atomistic view of the real inclusion process, while the use of water and acetonitrile was modeled to simulate the wash-out process. Future steps include the adoption of substituted βCDs in order to reach the desired control of the timescale of the release process, the analysis of solvation effects through explicit water molecules and possibly the modeling of a realistic nano-sponge.

## Figures and Tables

**Table 1:** Energetic ranking of the low-energy conformers of melatonin (MT). The numbers of MT structures correspond to the CCSD(T) ranking calculated by Fogueri et al. [47]. Values are in kcal/mol.

MT Structure N°				Relative Energy (Kcal/Mol)			
B3LYP	PBEh-3c	GFN2	GFNFF	B3LYP	PBEh-3c	GFN2	GFNFF
03	00	10	01	0.00	0.00	0.00	0.00
00	01	11	00	0.28	1.32	0.00	0.59
02	03	07	02	0.30	1.36	0.01	2.02
15	02	04	15	0.86	1.77	0.06	2.73
05	04	15	05	1.07	2.47	0.07	2.73
09	15	05	04	1.20	2.47	0.08	2.77
04	05	00	11	1.53	2.48	0.12	2.93
11	09	06	10	1.74	3.02	0.17	2.93
01	07	09	07	1.80	3.12	0.65	2.95
07	10	02	03	1.83	3.12	0.93	3.10
10	11	18	06	1.89	3.12	0.98	3.29
22	06	21	09	2.73	3.12	0.98	5.84
21	22	22	18	4.75	3.86	1.01	5.92
18	18	01	21	5.40	3.87	1.15	5.93
06	21	03	22	5.85	3.88	1.53	5.95

**Table 2:** Dimerization energy (ΔE), enthalpy (ΔH) and free energy (ΔG) of melatonin dimers (DIM), in all the studied conformations: Head–Head (HH), Head–Tail (HT), Tail–Tail (TT) and Indole–Indole (II). Values are in kcal/mol.

	GFN2			GFNFF			PBEh-3c			B3LYP
System	ΔE	ΔH	ΔG	ΔE	ΔH	ΔG	ΔE	ΔH	ΔG	ΔE
DIM-HH	−4.0	−2.7	9.2	−7.5	−5.3	7.2	−2.0	−9.6	5.2	−5.4
DIM-HT	−6.0	−4.6	6.7	−5.0	−3.3	10.4	−3.9	−11.2	3.2	−8.6
DIM-TT	−9.3	−8.0	3.9	−11.3	−9.0	3.1	−9.0	−16.3	−1.4	--
DIM-II	−15.0	−13.6	0.8	−16.6	−14.1	0.3	−11.9	−19.1	−2.4	−18.8

**Table 3:** Energy difference between stable CD isomers.

Method	Solvent	Energy Difference	CD2–CD1
PBEh-3c	Gas phase	ΔE	−4.4
B3LYP	Gas phase	ΔE	7.4
GFN2	Gas phase	ΔE	7.0
		ΔH	7.1
		ΔG	3.7
	Water	ΔE	−5.0
		ΔH	−5.0
		ΔG	−10.1
	Acetonitrile	ΔE	−4.4
		ΔH	−4.4
		ΔG	−8.7
GFNFF	Gas phase	ΔE	5.3
		ΔH	4.6
		ΔG	0.7
	Water	ΔE	−5.0
		ΔH	−5.0
		ΔG	−10.1
	Acetonitrile	ΔE	−4.4
		ΔH	−4.4
		ΔG	−8.7

**Table 4:** Complexation energy (with (ΔE_disp_) and without (ΔE_noD_) dispersive contribution) calculated at the xTB-GFN2 optimized geometries with different methods. Values are in kcal/mol.

Method	ΔE_disp_	ΔE_noD_
GFN2	−30.3	−13.3
GFNFF	−41.3	−18.1
PBEh-3c	−34.5	−19.9
B3LYP	−35.3	−6.4

**Table 5:** Complexation energy (ΔE), enthalpy (ΔH) and free energy (ΔG). Values are in kcal/mol. Legend: MT = melatonin, βCD = β-cyclodextrin, GP = gas phase, W = water, ACN = acetonitrile.

Method	Structure	ΔE	ΔH	ΔG
PBEh-3c	EXT	−39.4	−38.5	−18.2
GFN2—GP	EXT	−30.3	−28.7	−11.0
	MT-βCD1	−25.6	−23.7	−6.3
	MT-βCD2	−39.5	−37.2	−16.9
	MT-βCD3	−25.3	−23.4	−4.9
GFN2—W	EXT	−12.5	−10.2	7.2
	MT-βCD1	−14.2	−12.5	6.4
	MT-βCD2	−15.4	−12.7	4.7
	MT-βCD3	−13.5	−11.1	7.2
GFN2—ACN	EXT	−7.4	−5.3	10.4
	MT-βCD1	−7.0	−4.9	11.9
	MT-βCD2	−7.5	−5.2	12.4
	MT-βCD3	−5.5	−4.3	11.4

## Supplementary Materials

Table S1: CCSD(T) vs. GFN2 ranking of the 52 melatonin conformers. In yellow, the 15 structures used for the benchmark. Table S2: Complexation energy (ΔE), enthalpy (ΔH) and free energy (ΔG). Values are in kcal/mol. Legend: MT = melatonin, βCD = β-cyclodextrin, GP = gas phase, W = water, ACN = acetonitrile. Figure S1: Superimposed optimized structures of melatonin dimer. Color legend: GFN2 blue, GFNFF red, PBEh-3c yellow, B3LYP pink. Figure S2: Computed spectra for the Indole–Indole dimer at PBEh-3c (blue line) and GFN2 (orange line). Figure S3: Top and side view of βCD. The symmetry irreducible atoms are in ball and stick. Atom color: H white, C grey, O red. Figure S4: Superimposed optimized structures of melatonin dimer. Color legend: GFN2 blue, GFNFF red, PBEh-3c yellow, B3LYP pink. Figure S5: H bond evolution during the molecular dynamics simulation on the MT/βCD complex. Figure S6: Selected snapshots of the molecular dynamics simulation in implicit water solvation of the MT/βCD complex. Figure S7: Selected snapshots of the molecular dynamics simulation in implicit acetonitrile solvation of the MT/βCD complex.
