# Supplementary material for: On the Interactions of Melatonin/β-Cyclodextrin Inclusion Complex: A Novel Approach Combining Efficient Semiempirical Extended Tight-Binding (xTB) Results with Ab Initio Methods
Source: Molecules. 2021 Sep 28;26(19):5881. doi: 10.3390/molecules26195881 (PMC8512077; doi:10.3390/molecules26195881)
Supplement: Supplementary file 1 [file molecules-26-05881-s001.zip › molecules-1321206-supplementary.pdf]

Supplementary Materials

On the Interactions of Melatonin/β-Cyclodextrin Inclusion Complex: A Novel Approach Combining Efficient Semiempirical Extended Tight-Binding (xTB) Results with Ab Initio Methods

Riccardo Ferrero [1], Stefano Pantaleone [1,2], Massimo Delle Piane [3], Fabrizio Caldera [1], Marta Corno [1,*], Francesco Trotta [1] and Valentina Brunella [1,*]

[1] Dipartimento di Chimica and Nanostructured Interfaces and Surfaces (NIS) Centre, Università degli Studi di Torino, via P. Giuria 7, IT-10125 Torino, Italy; riccardo.ferrero@unito.it (R.F.); stefano.pantaleone@unito.it (S.P.); fabrizio.caldera@unito.it (F.C.); francesco.trotta@unito.it (F.T.)

[2] Dipartimento di Chimica, Biologia e Biotecnologie, Università degli Studi di Perugia, Via Elce di Sotto 8,
I-06123 Perugia, Italy

[3] Politecnico di Torino, Department of Applied Science and Technology (DISAT), Corso Duca degli
Abruzzi, 24, 10129 Torino, Italy; massimo.dellepiane@polito.it (M.D.P.)

* Correspondence: marta.corno@unito.it (M.C.); valentina.brunella@unito.it (V.B.)

Table S1. CCSD(T) vs GFN2 ranking of the 52 melatonin conformers. In yellow the 15 structures used for the benchmark.

CCSD(T)	GFN2
0	10
1	11
2	7
3	4
4	15
5	5
6	0
7	6
8	9
9	2
10	18
11	21
12	22
13	1
14	3
15	19
16	8
17	12
18	14
19	26
20	31
21	32
22	13
23	29
24	36

25	27
26	20
27	23
28	25
29	30
30	40
31	39
32	35
33	33
34	37
35	43
36	42
37	17
38	34
39	24
40	28
41	16
42	46
43	38
44	41
45	45
46	44
47	47
48	49
49	48
50	50
51	51

Table S2. Complexation energy (ΔE), enthalpy (ΔH), and free energy (ΔG). Values are in kcal/mol. Legend: MT = melatonin, βCD = β-cyclodextrin, GP = gas phase, W = water, ACN = acetonitrile.

Method	Structure	ΔE	ΔH	ΔG
PBEh-3c	EXT	-39.4	-38.5	-18.2
GFNFF - GP	EXT	-30.3	-28.7	-11.0
	MT-βCD1	-44.3	-39.9	-21.2
	MT-βCD2	-41.2	-38.0	-19.2
	MT-βCD3	-40.6	-36.3	-16.7
GFNF - W	EXT	-39.4	-35.8	-17.2
	MT-βCD1	-27.1	-23.5	-5.7
	MT-βCD2	-26.9	-23.6	-5.5
	MT-βCD3	-24.4	-21.1	-3.8
GFNFF - ACN	EXT	-24.8	-21.8	-4.4
	MT-βCD1	-32.3	-26.9	-6.4
	MT-βCD2	-19.5	-16.0	2.9
	MT-βCD3	-12.4	-9.9	7.4

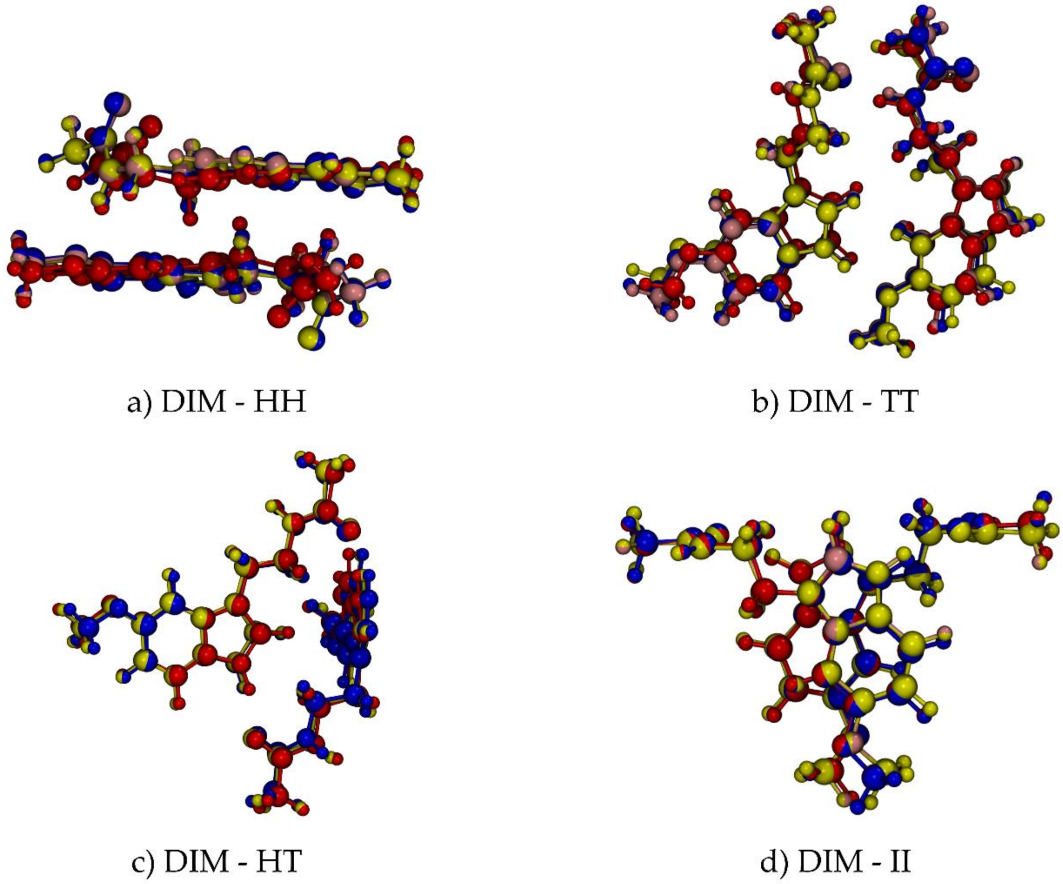

Figure S1. Superimposed optimized structures of melatonin dimer. Colors legend: GFN2 blue, GFNFF red, PBEh-3c yellow, B3LYP pink.

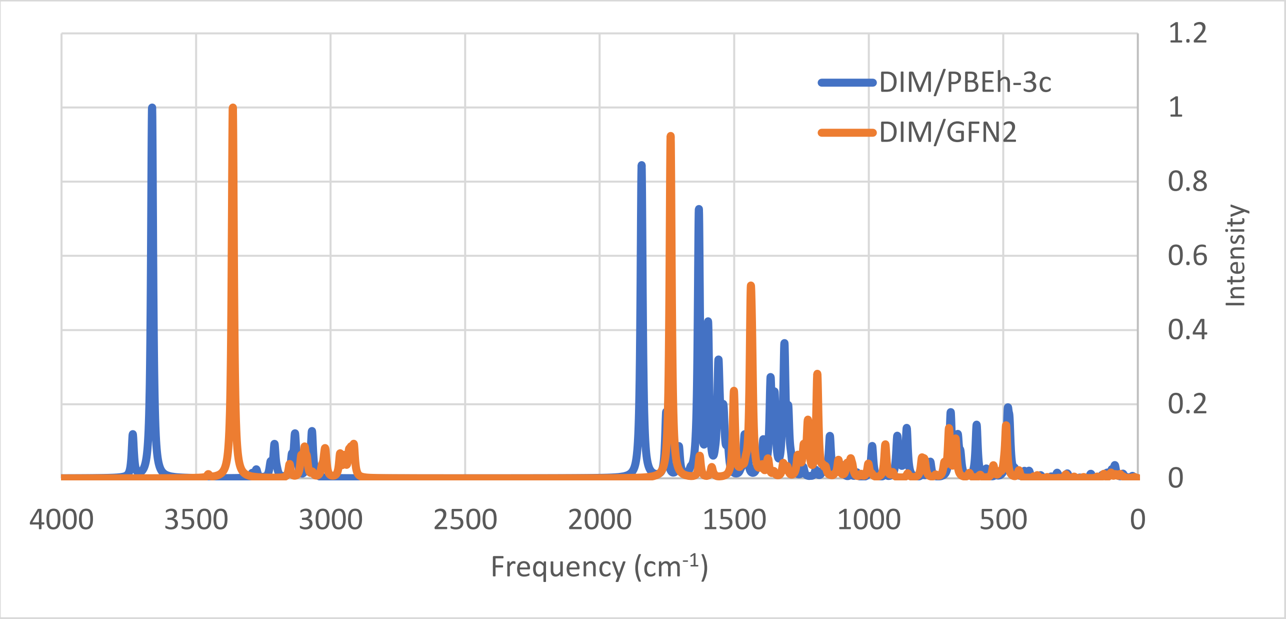

Figure S2. Computed spectra for the Indole-Indole dimer at PBEh-3c (blue line) and GFN2 (orange line).

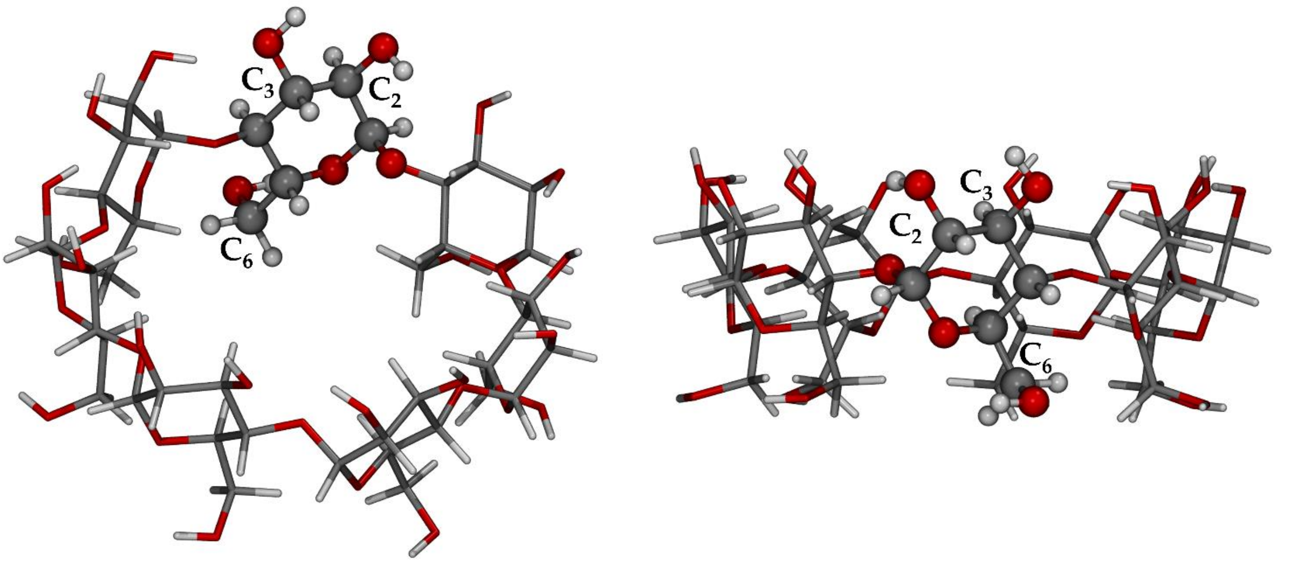

a) βCD – top view

b) βCD – side view

Figure S3. Top and side view of βCD. The symmetry irreducible atoms are in ball and stick. Atom color: H white, C grey, O red.

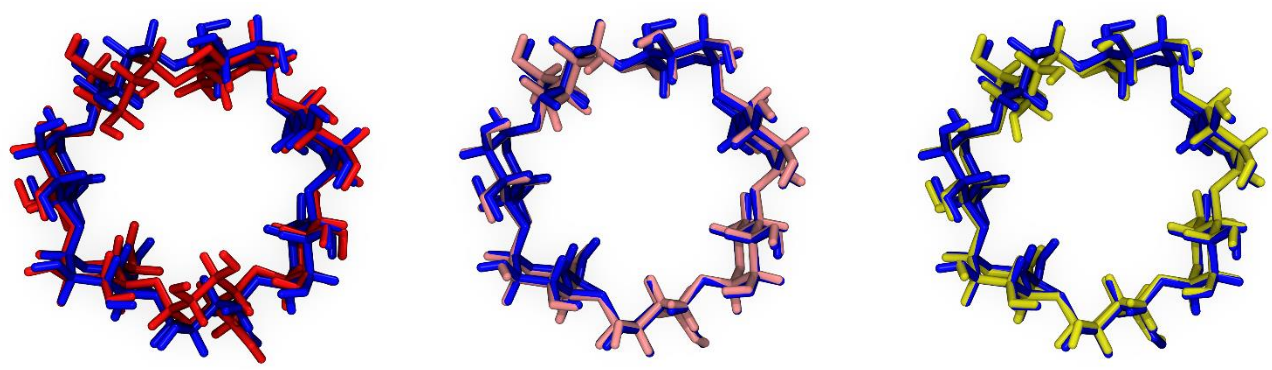

a) GFN2/GFNFF

b) GFN2/B3LYP

c) GFN2/PBEh-3c

Figure S4. Superimposed optimized structures of β-cyclodextrin. Colors legend: GFN2 blue, GFNFF red, PBEh-3c yellow, B3LYP pink.

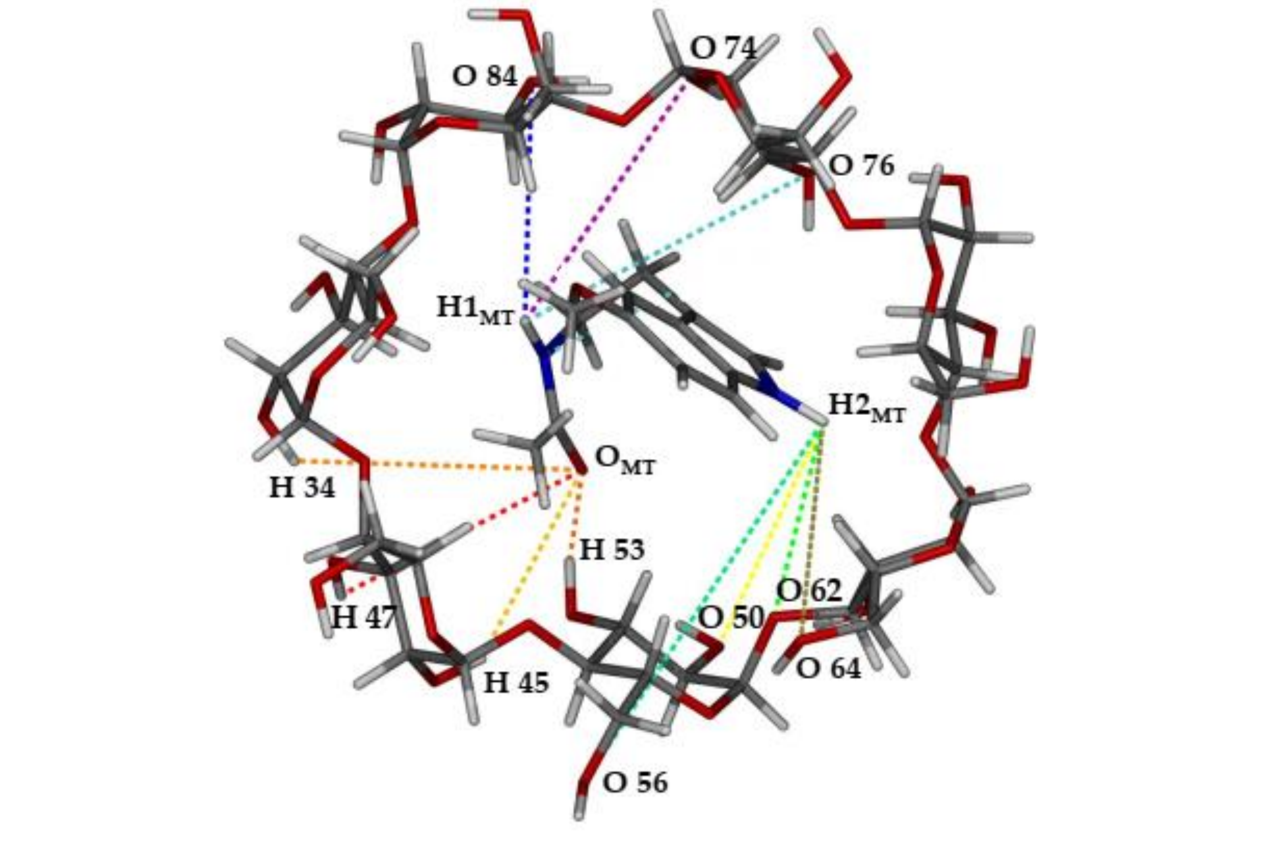

a) Starting point

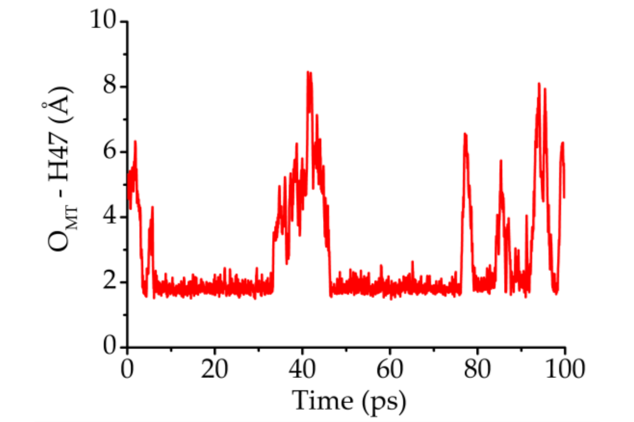

b) H47

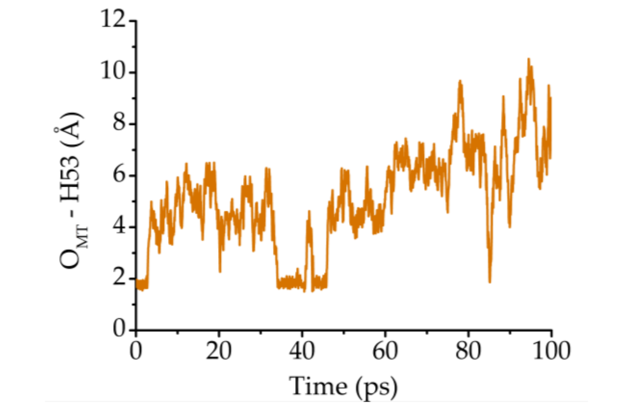

c) H53

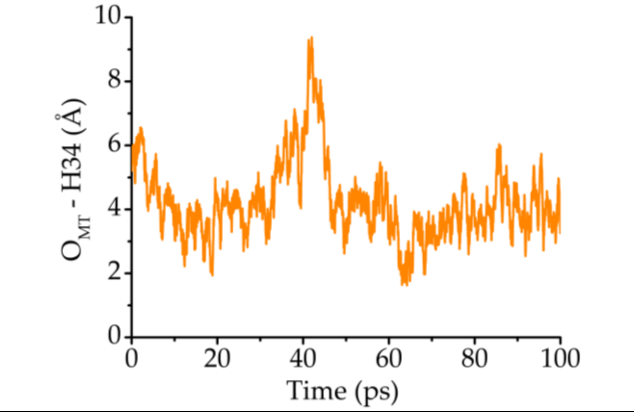

d) H34

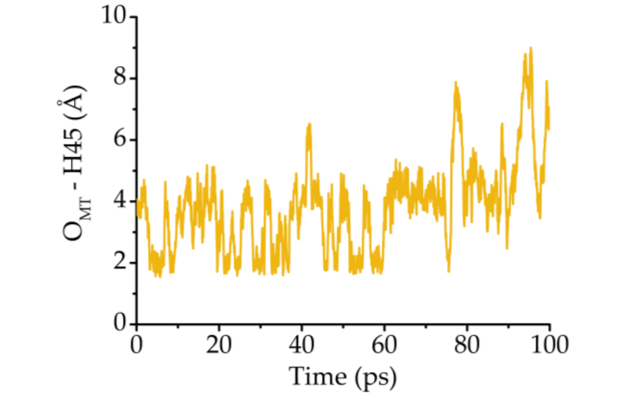

e) H45

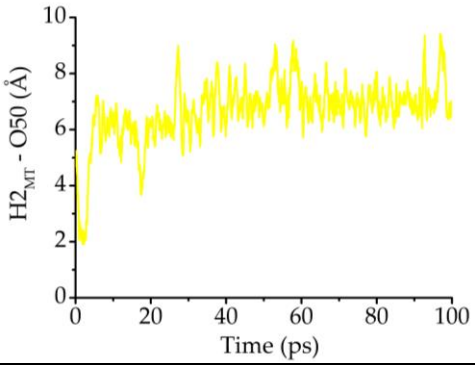

f) O50

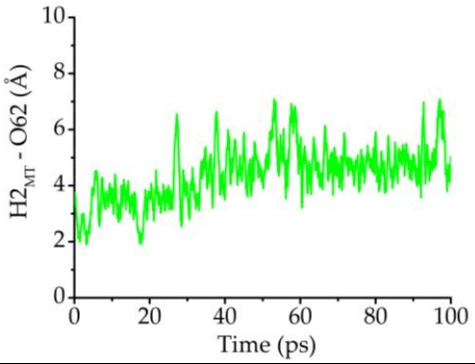

g) O62

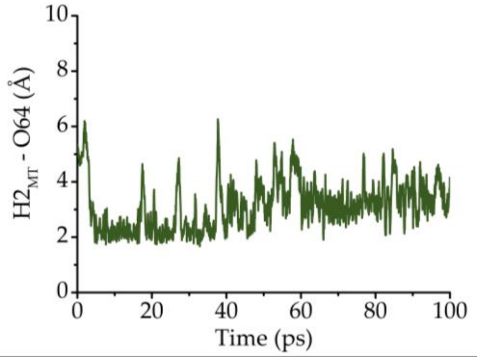

h) O64

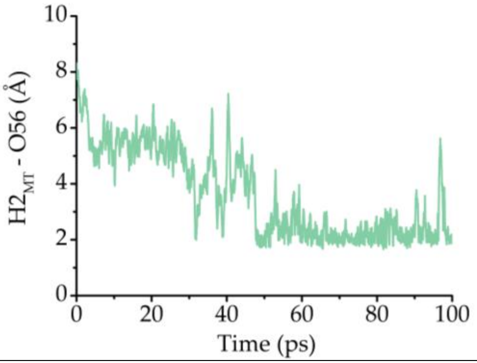

i) O56

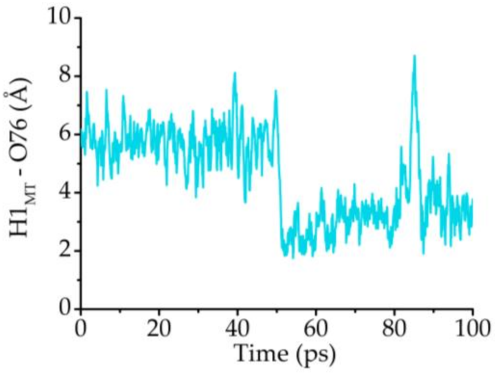

j) O76

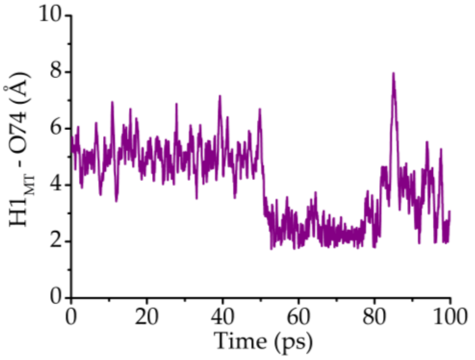

k) O74

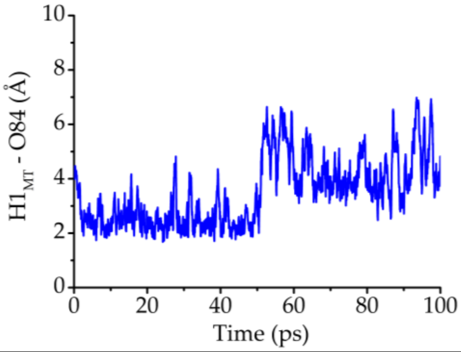

l) O84

Figure S5. H bond evolution during the molecular dynamic simulation on the MT/βCD complex.

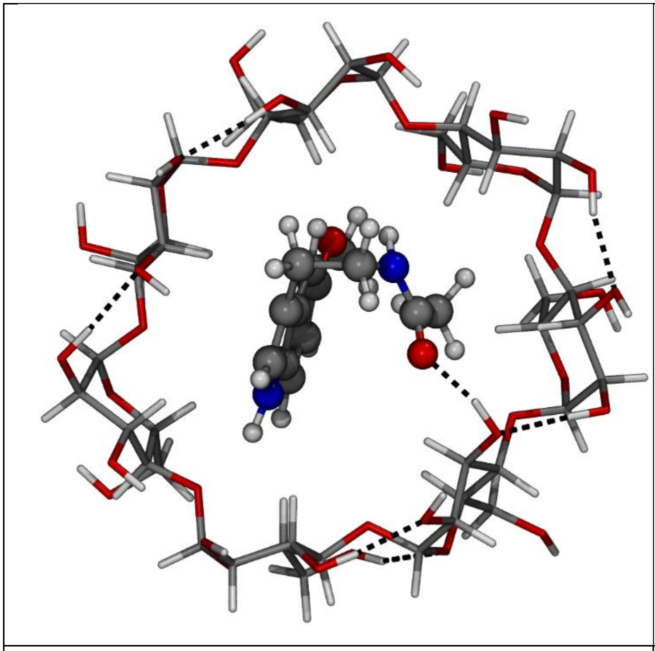

a) 0 ps - top

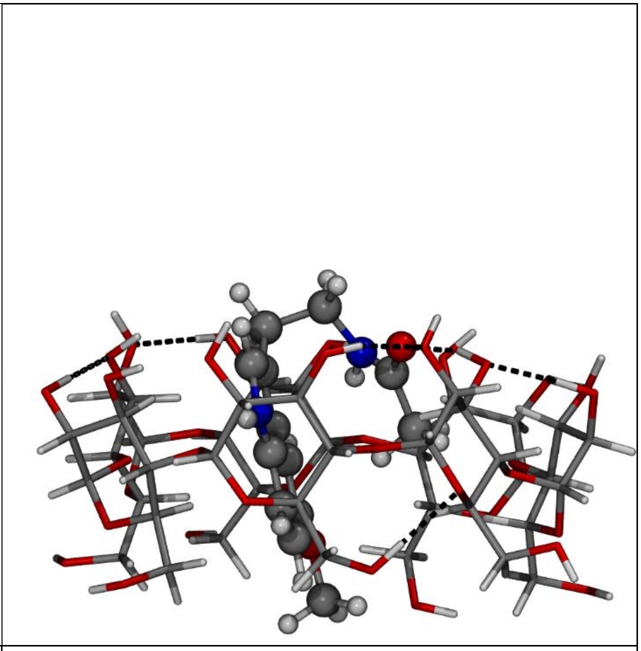

b) 0 ps - side

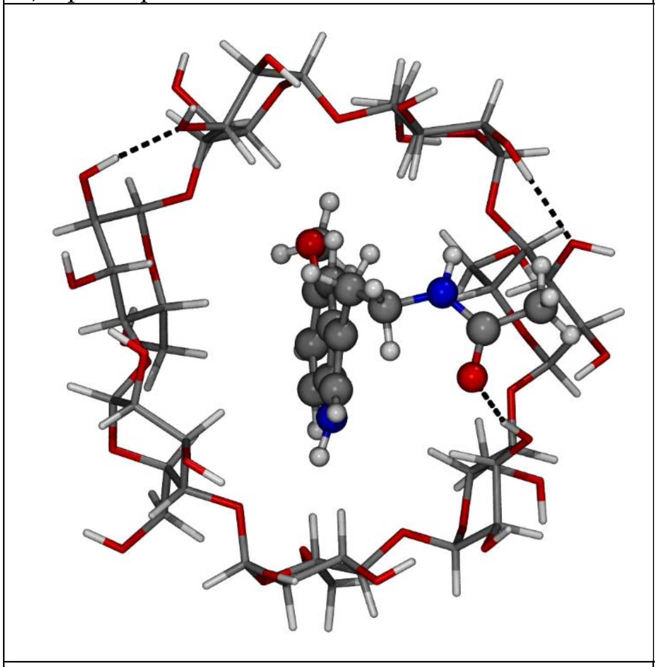

c) 10 ps -top

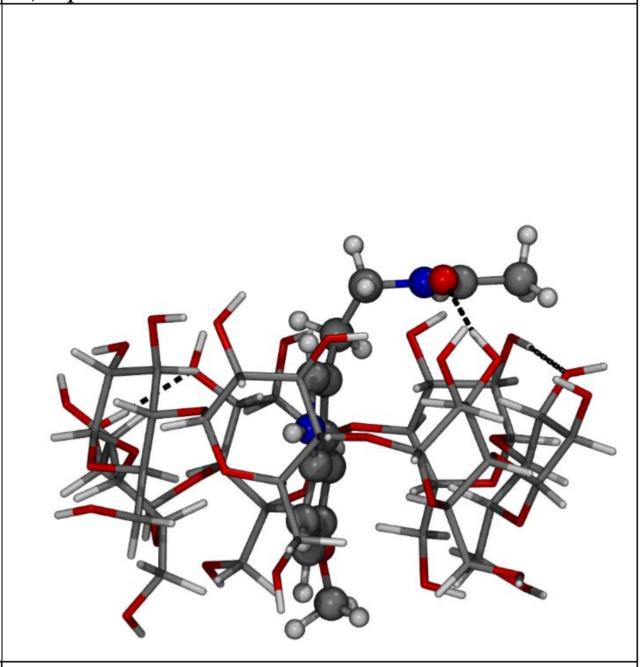

d) 10 ps -side

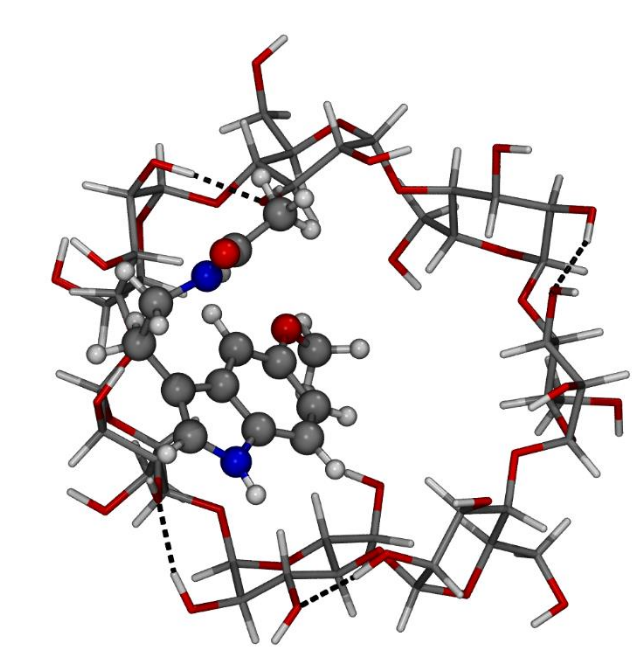

e) 25 ps – top

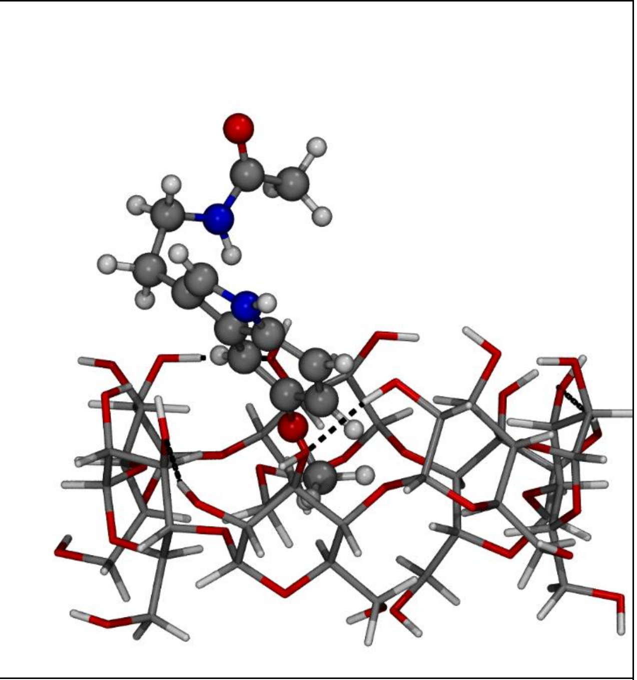

f) 25 ps - side

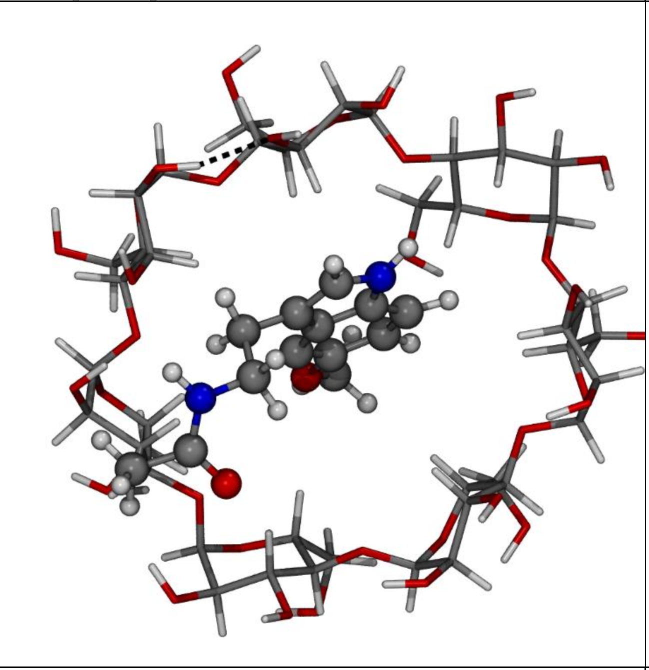

g) 50 ps – top

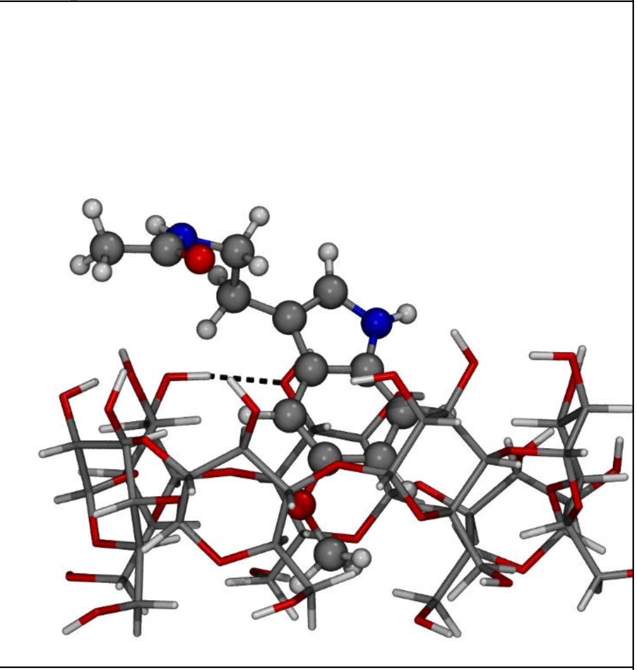

h) 50 ps – side

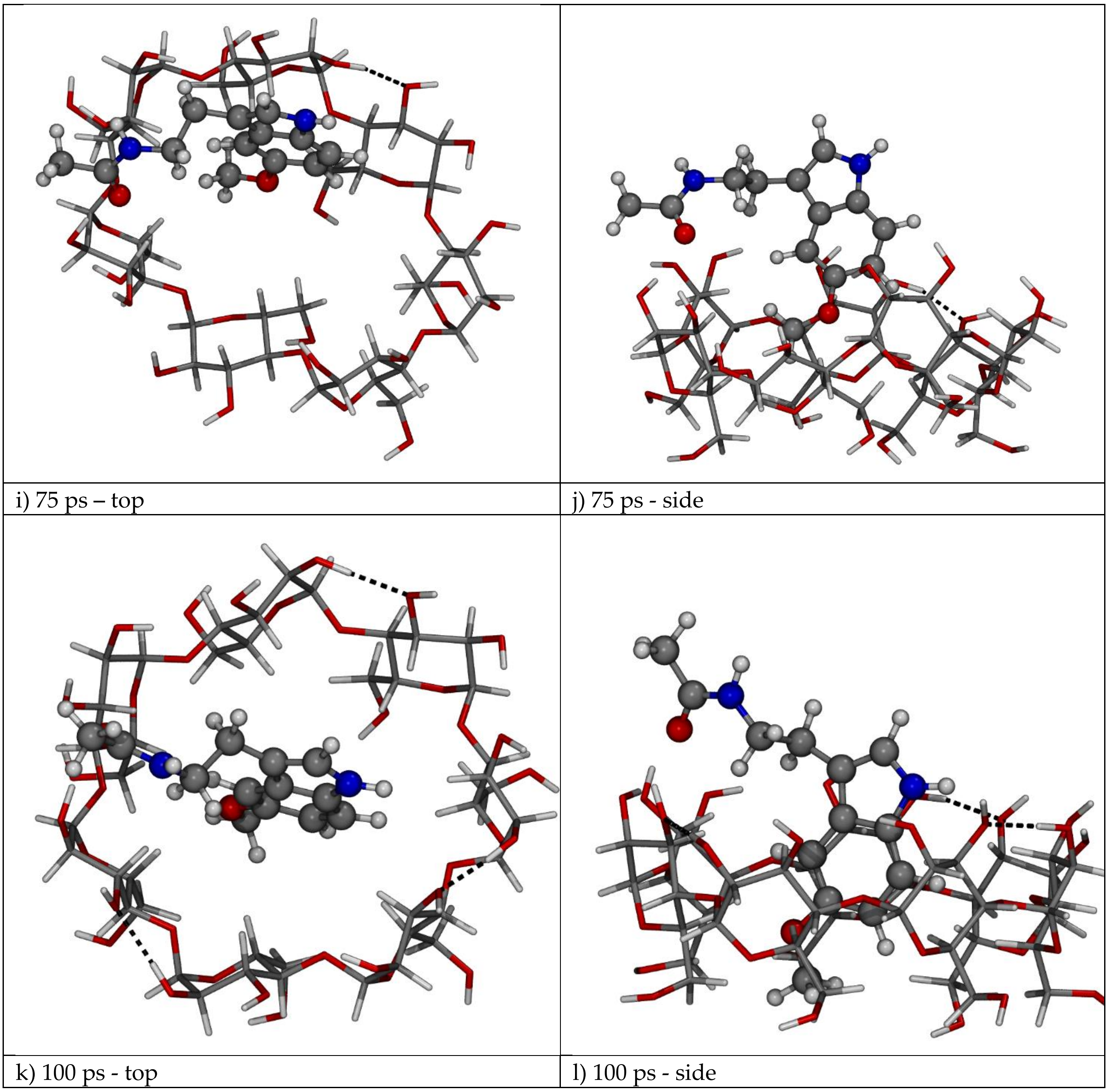

i) 75 ps – top
j) 75 ps - side
k) 100 ps - top
l) 100 ps - side

Figure S6. Selected snapshots of the molecular dynamic simulation in implicit water solvation of the MT/βCD complex.

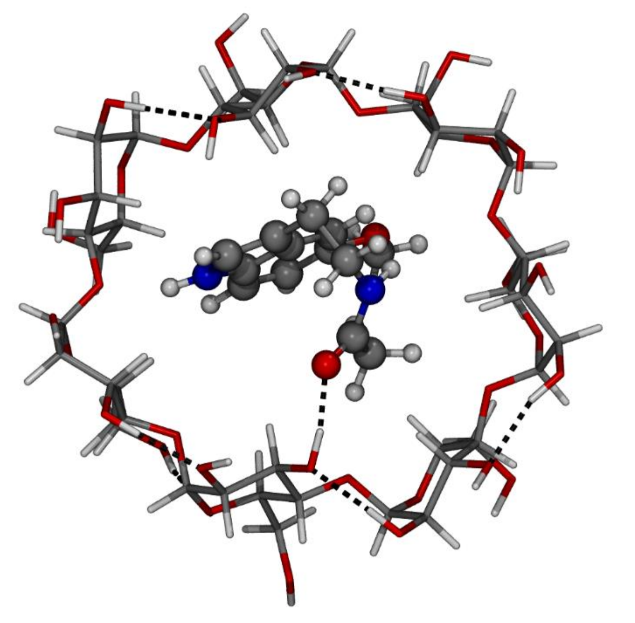

a) 0 - top

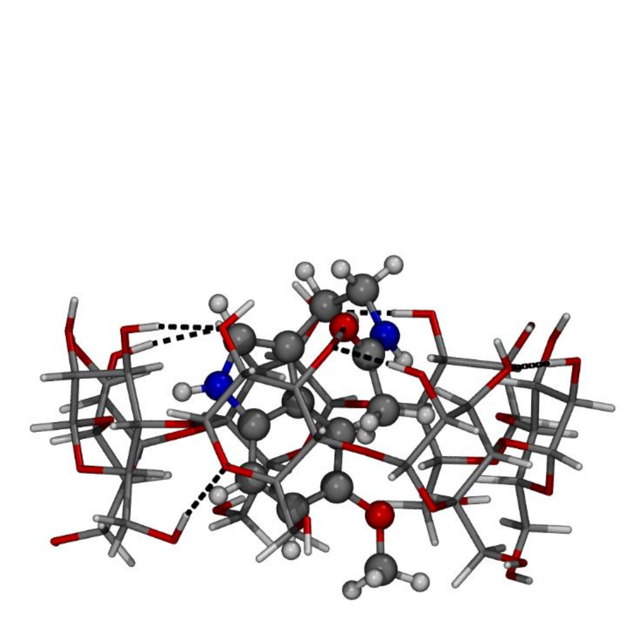

b) 0 – side

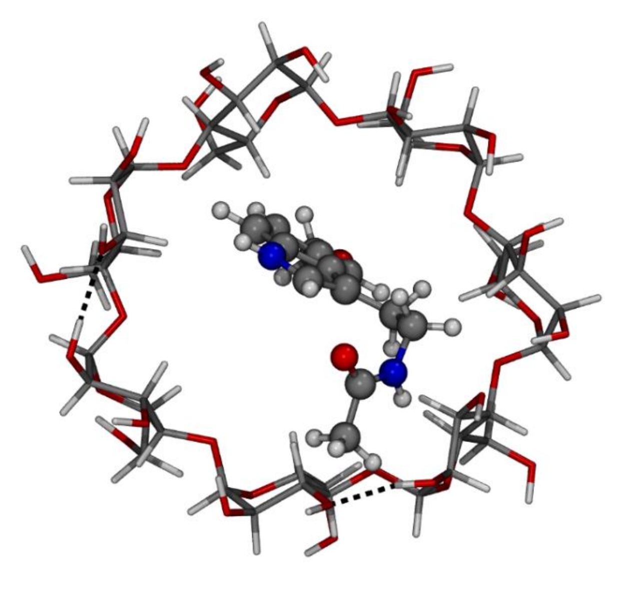

c) 10 ps – top

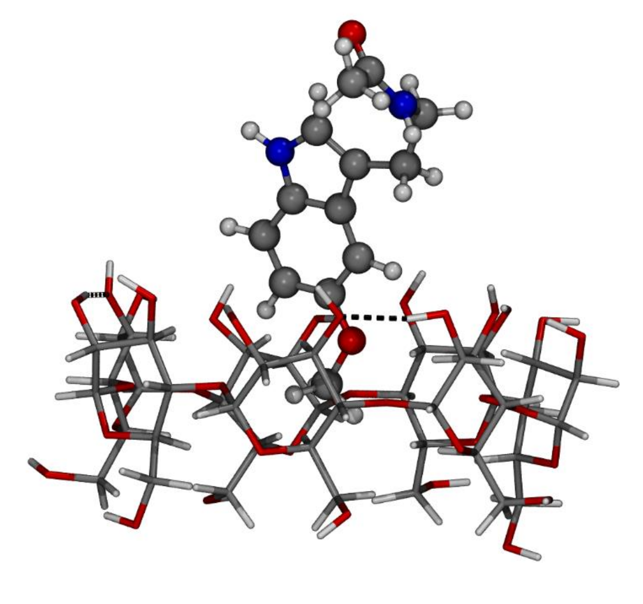

d) 10 ps - side

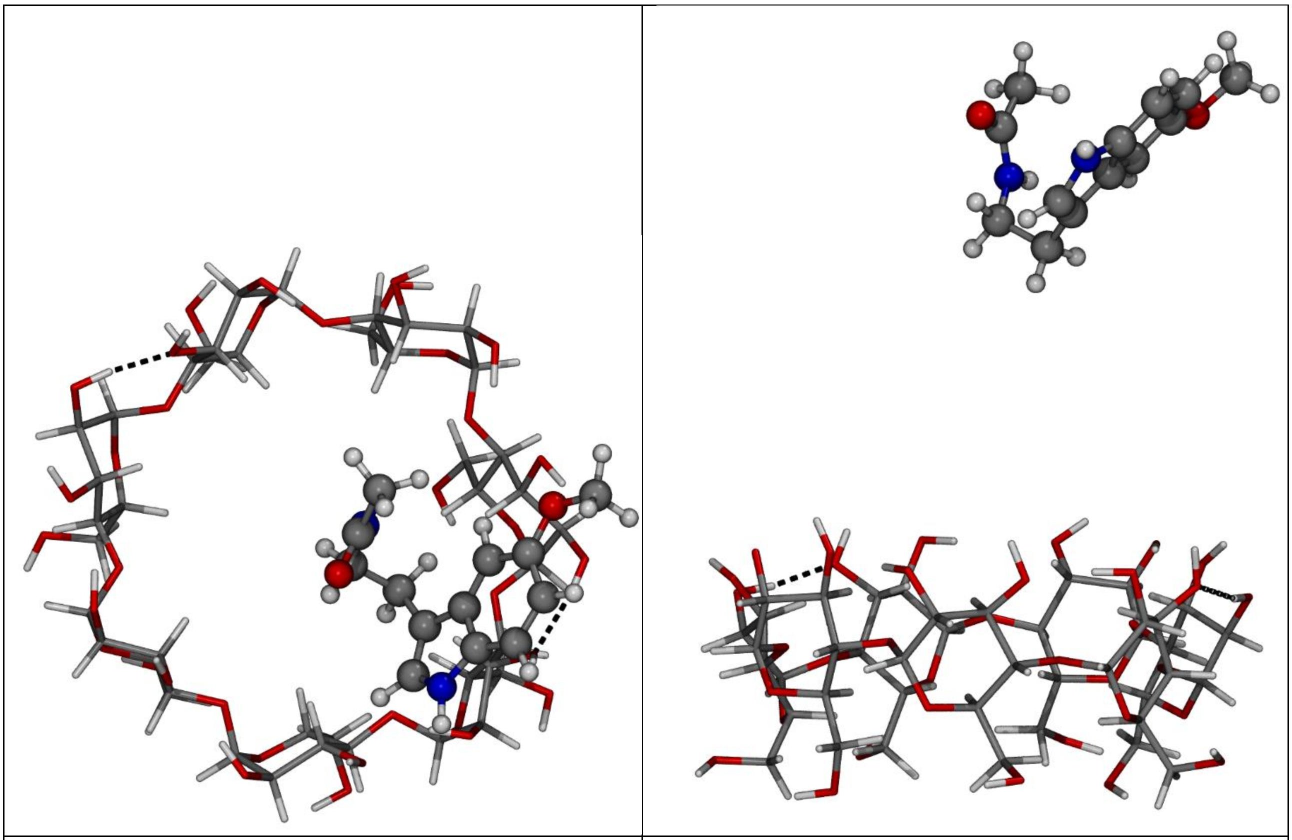

e) 15 ps - top

f) 15 ps - side

Figure S7. Selected snapshots of the molecular dynamic simulation in implicit acetonitrile solvation of the MT/βCD complex.
